# Potential Implications of Climate Change on *Aegilops* Species Distribution: Sympatry of These Crop Wild Relatives with the Major European Crop *Triticum aestivum* and Conservation Issues

**DOI:** 10.1371/journal.pone.0153974

**Published:** 2016-04-21

**Authors:** Marie-France Ostrowski, Jean-Marie Prosperi, Jacques David

**Affiliations:** 1 Institut National de la Recherche Agronomique, Unité Mixte de Recherche Amélioration Génétique et Adaptation des Plantes, Montpellier Supagro, France; 2 Montpellier Supagro, Unité Mixte de Recherche Amélioration Génétique et Adaptation des Plantes, Montpellier Supagro, France; Murdoch University, AUSTRALIA

## Abstract

Gene flow from crop to wild relatives is a common phenomenon which can lead to reduced adaptation of the wild relatives to natural ecosystems and/or increased adaptation to agrosystems (weediness). With global warming, wild relative distributions will likely change, thus modifying the width and/or location of co-occurrence zones where crop-wild hybridization events could occur (sympatry). This study investigates current and 2050 projected changes in sympatry levels between cultivated wheat and six of the most common *Aegilops* species in Europe. Projections were generated using MaxEnt on presence-only data, bioclimatic variables, and considering two migration hypotheses and two 2050 climate scenarios (RCP_4.5_ and RCP_8.5_). Overall, a general decline in suitable climatic conditions for *Aegilops* species outside the European zone and a parallel increase in Europe were predicted. If no migration could occur, the decline was predicted to be more acute outside than within the European zone. The potential sympatry level in Europe by 2050 was predicted to increase at a higher rate than species richness, and most expansions were predicted to occur in three countries, which are currently among the top four wheat producers in Europe: Russia, France and Ukraine. The results are also discussed with regard to conservation issues of these crop wild relatives.

## Introduction

Spontaneous hybridization between cultivated species and their wild relatives is a common phenomenon. From the standpoint of the wild relatives, when fertility is not completely lost, hybridization can lead to the incorporation of cultivated alleles in their gene pool. These events can have important evolutionary consequences, from increased extinction risk to increased weediness [1–3], which is in turn associated with invasiveness [4]. Over the last decades, much attention has been focused on crop-to-weed hybridization as a potential avenue for the escape of crop transgenes into natural populations [5]. This particular concern has increased the need to gain further insight into the specific risks of gene flow associated with major crops worldwide.

Levels of gene flow between crop species and their wild relatives are highly variable, depending on genetic, environmental and spatiotemporal factors [1]. Some relatives can be more prone than others to crossbreeding and/or to expressing hybrid fertility due to genetic and environmental determinants. However, physical proximity (sympatry) and flowering overlap are the necessary spatiotemporal conditions for hybridization and consecutive gene flow to occur.

Together with the allotetraploid durum wheat (*Triticum turgidum durum*, AABB, 2n = 4x = 28 chromosomes), the allohexaploid bread wheat (*Triticum aestivum L*., AABBDD, 2n = 6x = 42) is one of the major crops worldwide and the most important in Europe. On the evolutionary time scale, these cultivated species arose recently from hybridization events involving *Triticum* species and diploid species from the *Aegilops* genus [6]. The *Aegilops* genus comprises 22 species, among which only 10 are diploid, with the other 12 being allotetra (2n = 4x = 28) or allohexaploid (2n = 6x = 42). This high number of successful hybrid (amphiploid) speciation events stresses the importance of interspecific crossbreeding in their taxonomic group, i.e. Triticeae. In Europe, 12 *Aegilops* species are present, with the most widespread being *Ae*. *biuncialis* Vis., *Ae*. *cylindrica* Host, *Ae*. *geniculata* Roth, *Ae*. *neglecta* Req. ex Bertol., *Ae*. *triuncialis* L. and *Ae*. *ventricosa* Tausch [7]. All of these species are recent allopolyploids that spontaneously hybridize with wheat, yet they are predominantly selfing species. Wheat is cultivated in all European countries where *Aegilops* species are present, and they can have synchroneous flowering with wheat [8]. Like wheat, *Aegilops* species are annual grasses that can grow in tufts by tillering. However, tillering can be asynchroneous in *Aegilops*, which increases the total flowering window of these species as compared to wheat.

Spontaneous wheat x *Aegilops* hybrids were first described in the 19^th^ century [6]. They generally possess the tough rachis of wheat and spike morphology of the *Aegilops* spp. Natural hybrids are typically observed in open areas between roads and adjacent wheat fields, or next to fields where wheat had been cultivated the previous year. The main ecological features of *Aegilops* species may be summarized by hot and dry summers and winter rainfall. However, variations in ecological tolerance exist from species to species [7]. Some *Aegilops* species are typical colonizers that are able to rapidly invade new territories. *Aegilops biuncialis*, *Ae*. *cylindrica*, *Ae*. *geniculata*, and *Ae*. *triuncialis* are considered as the best colonizers and develop large stands [7]. Although invasive, *Aegilops* species are not considered as noxious weeds in Europe, as opposed to the United States where *Aegilops cylindrica* and *Ae*. *triuncialis* are both present. First introduced in the US about a century ago, they have since colonized large areas and are still progressing [9–10]. Whereas *Ae*. *triuncialis* is mainly weedy in Californian pastures where it diminishes the feed quality for cattle [11], *Ae*. *cylindrica* occupies wheat fields and field margins in western and central states, causing millions of US dollars in yield losses [12] and it can hybridize at rates as high as 8% on a per field basis [13].

The genomic constitution of *Aegilops* species leads to differential affinities with wheat chromosomes [6]. *Aegilops cylindrica* and *Ae*. *ventricosa*, in particular, both share a common D genome with bread wheat originating from the parental diploid species *Ae*. *tauschii*. This close relatedness increases chromosome pairing at meiosis and recombination at the hybrid stage, which may facilitate consecutive introgressions [14]. However, there is considerable genetic variation for inter-specifique chromosome pairing, depending on the wheat cultivar and the wild relative species [15].

Only recently some evidence of wheat introgressions in natural *Aegilops* populations—beyond the F1 hybrid stage—was published [16–18]. The recipient populations were found in Europe, where the *Aegilops* species and cultivated wheat have a long history of co-existence. None of the reported recipient species (*Ae*. *triuncialis*, U^t^U^t^C^t^C^t^ and *Ae*. *neglecta*, U^n^U^n^X^n^X^n^) shares a parental species genome with wheat (but see also [19] for Near East accessions of *Ae*. *peregrina* whose S genome is close to the wheat B genome). Although breeders have been highly successful in introgressing *Aegilops* choromosomic regions in wheat for agronomic purposes, there is little evidence of successful natural introgression of cultivated wheat genes in *Aegilops* genomes. However, scarceness cannot yet be considered as conclusive with regard to gene flow levels from cultivated wheat to any wild *Aegilops* relatives. Ploidy levels and the recent common ancestry of *Triticum* and *Aegilops* genera are making introgressions very challenging to assess using molecular approaches. Combined with other strategies, investigations on sympatry levels thus represent a key way to gain insight into species specific risks of gene flow.

As a consequence of climatic change, shifts in the distribution of many wild species have already been observed [20–22] and this trend is expected to continue [23]. Environmental niche modeling (ENM) approaches are now being applied to clarify a variety of questions regarding the impact of climate change on the biodiversity, range, history and evolution of species [24–29], including cultivated and some wild related species [30–32]. To our knowledge, however, the possible impact these changes on crop-to-weed gene flow has not yet been addressed. Provided that wild related species will shift and/or expand their range under climatic pressure, sympatry levels with crops can be anticipated to change as well. Here we propose an ENM assessment of current and projected future distributions of the six most common *Aegilops* species in Europe which are closely related to wheat. Distribution models were generated using MaxEnt (v. 3.3.3; https://www.cs.princeton.edu/~schapire/maxent/), one of the most effective techniques for the analysis of presence-only data [33–34]. It works by contrasting the range of environments associated with species occurrences with the range of environments across the considered landscape [35–36]. Current distributions represent geographical locations where climatic conditions are predicted to be suitable for the species by the chosen correlative model. Future distributions are projected under the assumption that current modeled distributions reflect species climatic preferences, which will be retained under climate change. This assumption is fairly well supported by a number of studies that have provided evidence of the conservatism of ecological niches over time [37]. Future distributions were projected using two 2050 climate scenarios (RCP 4.5 and 8.5) averaged over 16 models. We further considered two contrasted migration rates (zero and universal) in calculating future pixel losses (zero migration) and pixel net gains (universal migration: gains minus losses) at both global and regional scales. Like crop wild relatives, *Aegilops* species are not only potential introgression recipients but also an invaluable source of genes for the improvement of cultivated wheats [38]. These future distributions were therefore examined in the context of eventual changes in gene flow opportunity—assuming that no major change in the wheat production surface area will occur in Europe by 2050—but also discussed from the genetic resource conservation perspective.

## Methods

### Data sources

Our samples combined geo-referenced presence-only data from the Global Biodiversity Information Facility (Gbif, http://www.gbif.org/), five French botanical institutes (Conservatoires Botaniques Nationaux) and additional observation data kindly provided by M. W. van Slageren (pers. com.; details in the S1 Table and S1 Appendix; complete dataset in the S1 File). Data were cleaned by excluding records that were derived from a different country than stated by the collector or outside the documented range of the species (Diva-gis, http://www.diva-gis.org/; Google Earth, http://www.google.com/intl/fr/earth/). The modeling was performed at a coarse-grained scale in order to buffer both uncertainties on point coordinates and differences in sampling intensity across regions. Accordingly, we kept a single occurrence record per 30 arc min grid cell, covering at best the documented Old World range of each species [7]. In the MaxEnt framework, a background grid is required to define the coordinates to be used when constructing density curves for the environmental variables during the training phase [36]. These grids were adjusted on a species specific basis, excluding regions where species were never reported, according to the recommendations [36]. In addition, because occurrence collections are often spatially biased in favour of easily accessed areas, these grids were assembled using the target group method [39] (target group: Angiospermae). This method excludes cells presumably unvisited by the botanists who contributed to the collections so as to reduce eventual sampling bias over the environmental space of the models [39–40] (grids and details available in the S1 Appendix).

The climate was described using 19 Bioclim variables derived from monthly temperature and precipitation data [41**–**42]. These gridded variables for current and future climatic conditions were directly accessed via the WorldClim data portal and downloaded at the 10 and 30 arc min spatial resolution (http://www.worldclim.org/; variables are described in the S2 Appendix). Current climate raw data were interpolations from weather station data spanning the 1950 to 2000 period [43]. Future IPPC_5_ climate data for 2050 (2041–2060 averages) were derived from General Circulation Models. The calibration and downscaling method is Available http://www.worldclim.org/. For future climate, the central trends of climate models were preferred over those derived from single models. Therefore, grid data were averaged over 16 models for which simulations were consistently available for two chosen Representative Concentration Pathways (RCPs listed in S2 Table). RCPs correspond to four trajectories (time series) in anthropogenic radiative forcing agents (mainly greenhouse gas concentration) adopted by the Intergovernmental Panel on Climate Change (IPCC) in its fifth Assessment Report (AR5) in 2014. IPCC replaced the former SRES scenarios adopted for the Fourth Assessment (AR4). These time series served as climate model input. RCPs were named after the four IPCC selected limits in radiative forcing increases attained by 2100: +2.6 Wm^-2^, +4.5 Wm^-2^, +6 Wm^-2^ and +8.5 Wm^-2^. We used climate models derived from RCP_4.5_ and RCP_8.5_ which resulted in intermediate-low and worst case scenarios, respectively [44–45].

Wheat harvested area regional data per EU country were accessed via the Eurostat data portal (http://www.ec.europa.eu/eurostat). Non-EU country data originated mainly from the FAOstat data portal (http://www.faostat3.fao.org/). For Ukraine and Russia, regional data (oblast level) were derived from maps published by the FAS-USDA (http://www.pecad.fas.usda.gov). Russian data were found to be available for a single year and corresponded to the wheat sown area in 2004. Raw data were averaged within the time range [2000–2009], with variations depending on data availability. Averages were divided by the total area of the corresponding territorial unit and gridded at 10 arc min spatial resolution. Grid cells included in each unit were thus given the same fractional value and served as a constant proxy over time for the cultivated wheat encounter probability. Proxy values ranged from 0 to 0.27 depending on the administrative unit.

### Settings for model training

The geographically mapped results we used corresponded to the MaxEnt logistic output, which is best interpreted as a climatic suitability index for the species over the landscape [36]. Because we had no definite *a priori* about the relative importance of the climatic variables to discriminate presence points from background points across the six focal species, we used the same complete set of bioclimatic variables for all species (S2 Appendix). Once trained, the models were projected at higher resolution (10 arc min) and wider geographical extent for the current and 2050 future climate scenarios (S1 Appendix). The modeling options differing from the default settings in MaxEnt were as follows. In addition to the implemented background grids (bias grid option), models were run using a maximum number of iterations of 5000, with the extrapolate option turned on vs. off (see below) so as to restrict the projections to within the range of the climatic variable distributions used for training. Moreover, to better control over-fitting, we manipulated the beta multiplier of the pre-tuned regularization parameters of MaxEnt according to a previous study [46]. Increasing the default setting of this multiplier (β = 1) yields smoother response curves and therefore more general relationships [47]. Behind the scene, this act through an actual decrease in the number of fitted parameters. For each model, the beta value was chosen using the lowest AIC score corrected for sample size (ENM tools 1.4.1, [46]). The beta value was allowed to vary with an increment of 0.25, i.e. ranging from β = 1 to β = 5.

### Dealing with extrapolation

To evaluate the effect of extrapolation on the projected predictions, all models were run with and without the extrapolation option activated and then compared. Extrapolation was geographically structured and the extrapolated areas were wider in the 2050 projection grids than in the current ones. Although representing only a small fraction of the total number of the cells predicted to be suitable, we used raster layer algebra to exclude these cells as much as possible from the comparisons. For each model (species), cells associated with an extrapolated suitability value in the current climate projection grid were assigned a zero value in all projection grids obtained when allowing for extrapolation. In doing so, we avoided modifying cell values that were not extrapolations and excluded most of the extrapolated cells that were predicted to be suitable (possible commission errors). The remaining fraction is reported in the S3 Table.

### Evaluating the models

The absolute area under the ROC curve (AUC) value was previously found to be a poor criterion for evaluating ENM models, especially with presence-only data [48]. The significance of the association between occurrence data and climatic variables was therefore assessed using the one-tailed randomization test previously proposed by Raes & Steege [48]. To perform this test, a thousand pseudo-samples of the same size as the real dataset were randomly sampled in each species background grid using a custom R script (R v3.0.3; http://www.r-project.org/, {raster} and {base} packages). The AUC value of each random pseudo-sample was then estimated by MaxEnt using the same settings as for the selected model. This provided a null empirical distribution of AUC values associated with each particular combination of background and sample size. All six models were also run by performing ten-fold cross-validation, and the lowest AUC test value was compared to its corresponding null distribution. These AUC test values correspond to the AUC computed over an unused fraction of the real data when training the models (see Appendix 4 in [36]) Cross-validation experiments were also used to assess the prediction variability. This variability was summarized by calculating the difference in potential area of occupancy between the minimum and maximum statistical grids provided by MaxEnt.

### Estimates

The importance of the bioclimatic variables was examined using three summary statistics provided by MaxEnt [41]. All information and results relevant to this aspect are available in the S2 Appendix. The projected distributions were summarized via counts of suitable and unsuitable cells for each species. We used the maximum training sensitivity plus specificity logistic threshold to transform the continuous logistic index into binary predictions (0/1 scale) [49]. Summaries were produced for the full extent of the projection grid and for two geographical sub-units of comparable size: a European vs. non-European zone. The 2050 projections were compared to the current projections while considering two migration hypotheses: no migration and universal migration (eight combinations). Although real migration rates are unknown and more likely to be midway between these two extreme cases, this approach set boundaries for the predicted outcomes [26]. Newly suitable cells were assumed to be reachable under the universal migration hypothesis. The count of the 2050 suitable cells was therefore defined as C+G-L, where C is the count of the currently suitable cells, G is the 2050 cell gains, L is the 2050 cell losses and G-L is the 2050 net gains. Conversely, newly suitable cells were assumed to be unreachable under the no migration hypothesis. Consequently, the count of the (remaining) suitable cells was defined as C-L under the latter hypothesis. For each species, we quantified the potential area of occupancy as the number of climatically suitable cells over the total number of grid cells (filling estimates expressed in %). For each cell, potential species richness was estimated as the sum of the binary suitability index over the six species.

Sympatry levels between *Aegilops* species and cultivated wheat in the European zone were roughly estimated using a sympatry index. For each grid cell, this index was defined as the binary suitability index of each species times the constant proxy over time for the cultivated wheat encounter probability (proxy range: 0 to 0.27). To summarize the sympatry for each individual species, the index was summed over cells (species PSI) and this sum was also divided by the number of cells predicted to be suitable (species mean PSI). Importantly, because of the scale of the study and the lack of sufficient available abundance information, all models were run with the default prevalence parameter (τ). In doing so, we scaled the logistic suitability score based on the assumption that ‘typical’/‘average’ conditions at occurrence sites were associated with a climatic suitability of 0.5, for all six species [36]. Changing this parameter value results in changing the logistic suitability scores [50] but does not change the ranking of environments [36]. However, this implies that comparing continuous suitability scores between species can be particularly inappropriate because the ranking is species specific. For instance, one species might be very common at sites associated with, say, a suitability above 0.6, whereas another can be much less common for the same suitability level. Here, binary outputs provided a kind of a workaround, i.e. when summed over species to produce summary estimates, they could be interpreted in terms of species richness. However, the results must be gauged with the understanding that they do not take the differences in abundance of the six considered *Aegilops* species into account.

## Results

### Model evaluation

The β regulation parameter values yielding the best AIC_cor_ scores ranged from 1.25 to 3.75. The selected model for *Ae*. *triuncialis* and *Ae*. *cylindrica* had the highest number in fitted parameters (Table 1). As expected and explained in detail elsewhere [48], the AUC values were correlated with the prevalence of species specific occurrences in their respective backgrounds (see Table A1S2 in the S1 Appendix). However, for each model, the actual AUC value was greater than any value from the generated null distribution. The lowest ten-fold AUC test values were also greater or equal to (*Ae*. *cylindrica*) the maximum value of the null AUC distributions (Table 1). This indicated that correlations between species localities and the climatic predictor variables, as identified and interpolated by MaxEnt, deviated from random chance.

**Table 1:** Model selection and evaluation.

Species	β	AUC model	AUC null dist. range	*P*	AUC test	OR
*Ae*. *biuncialis*	3.50 (38)	0.83	0.53–0.63	<0.001	0.74	17%
*Ae*. *cylindrica*	1.25 (65)	0.83	0.61–0.71	<0.001	0.71	21%
*Ae*. *neglecta*	3.00 (42)	0.81	0.53–0.62	<0.001	0.76	26%
*Ae*. *geniculata*	3.00 (46)	0.78	0.53–0.59	<0.001	0.73	15%
*Ae*. *triuncialis*	1.75 (76)	0.76	0.53–0.60	<0.001	0.71	10%
*Ae*. *ventricosa*	3.75 (24)	0.88	0.55–0.69	<0.001	0.69	9%

The regularization parameter β corresponds to the value yielding the best AIC score corrected for sample size. AUC model and AUC null dist. correspond to the area under the ROC curve obtained when using the real sample for training each selected model and when using random pseudo-samples of the same size drawn from each background, respectively. Statistical significance (*P*) was assessed by determining the rank of the AUC model relative to AUC null dist. The AUC test value corresponds to the lowest value obtained on withheld data when performing ten-fold cross-validation. The omission rate (OR) of the selected model is also reported.

### Current projections

**Fig 1:**
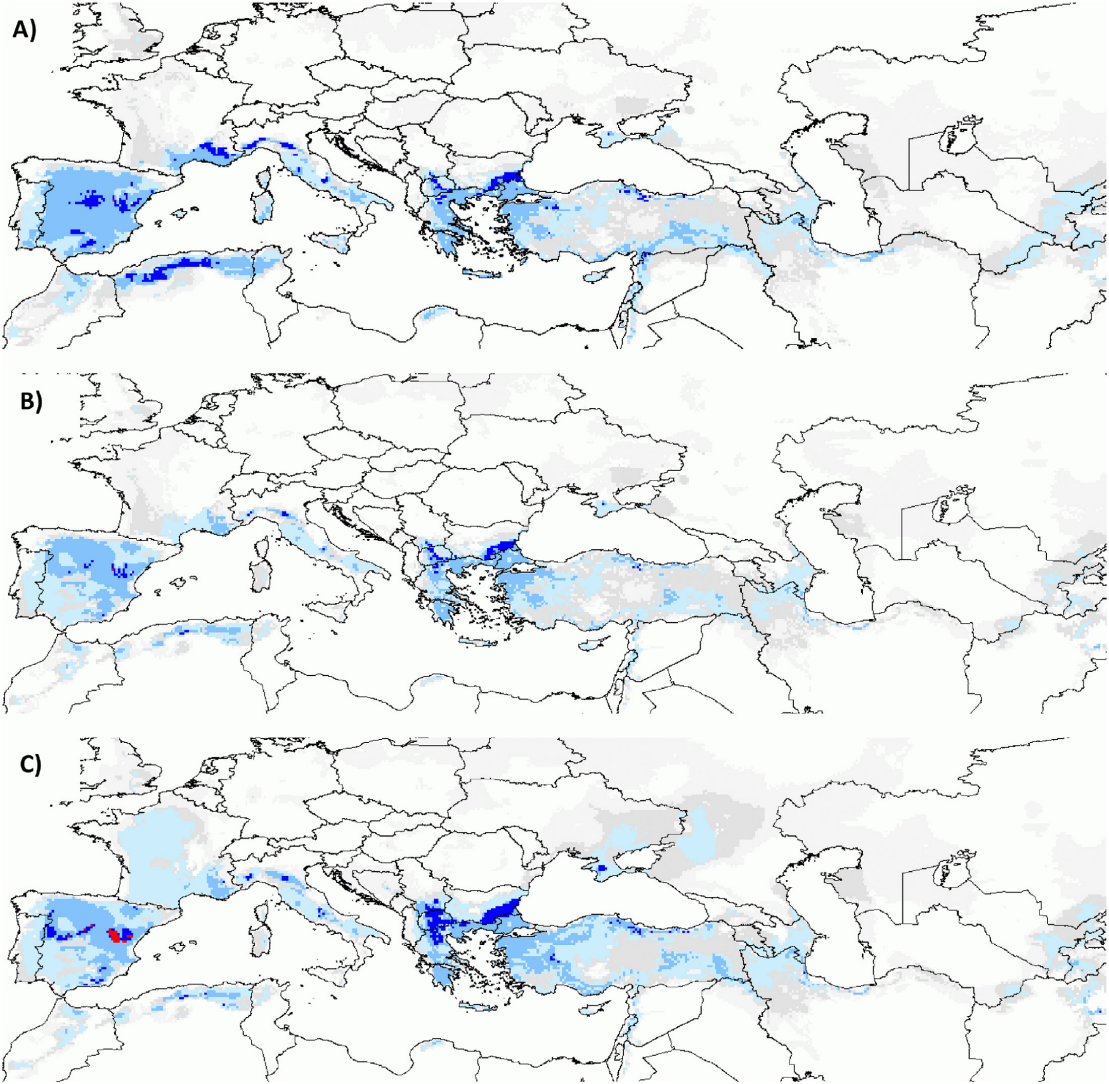
Potential species richness: RCP_4.5_. Predicted richness for **(A**) the current climate, **(B)** RCP_4.5_ under the no migration hypothesis and **(C)** RCP_4.5_ under the universal migration hypothesis. Light grey, dark grey, light blue, medium blue, dark blue and red correspond to cells predicted to be suitable for one to six *Aegilops* species.

Across the full extent of the current climate projection grid, the predicted richness (Fig 1) was 0.81 species per cell (Table 2). *Aegilops cylindrica* and *Ae*. *biuncialis* predicted distributions were the most eastward, whereas suitable conditions for *Ae*. *ventricosa*, *Ae*. *geniculata* and *Ae*. *neglecta* were found to be more westward (S1 Fig, Table 3). *Aegilops triuncialis* was associated with the widest potential range in longitude (S1 Fig). However, the predicted distributions of the two species bearing a D genome, *Ae*. *ventricosa* and *Ae*. *cylindrica*, were the most contrasted, i.e. the narrowest/most westward and the widest/most eastward, respectively (S1 Fig, Tables 2 and 3). *Aegilops cylindrica* was also characterized as having the most distinctive potential area of occurrence (hereafter PAO). For instance, 65% of its PAO did not overlap with that of any of the other species. However, *Ae*. *cylindrica* was also associated with the greatest prediction variability, as illustrated by the difference between the maximum and minimum filling estimate obtained for this species. This difference was also greater than that noted for the five other species for both 2050 projections (Table 4). When restricting calculations to the European zone, the PAO of *Ae*. *cylindrica* remained the largest, although *Ae*. *geniculata* took over the 2^nd^ rank from *Aegilops triuncialis* (S3 Table). In Europe, suitable areas for *Ae*. *geniculata* nearly completely overlapped the PAO of *Ae*. *ventricosa*, *Ae*. *biuncialis*, *Ae*. *triuncialis* and *Ae*. *neglecta*, in line with previously published distributions [7].

**Table 2:** Filling estimates, richness and predicted changes.

	Filling (%)	Change (%)			
		*UM*		*NM*	
	C	4.5	8.5	4.5	8.5
**Grid**					
*Ae*. *biuncialis*	11.0	24.0	25.3	-26.6	-36.5
*Ae*. *cylindrica*	23.9	16.5	11.0	-30.7	-37.7
*Ae*. *geniculata*	12.3	-0.6	-8.0	-19.3	-30.0
*Ae*. *neglecta*	8.4	21.8	15.4	-16.3	-25.9
*Ae*. *triuncialis*	18.7	8.8	10.2	-16.5	-21.0
*Ae*. *ventricosa*	6.3	-59.7	-74.3	-60.5	-74.9
Richness (x100)	**80.6**	**7.7**	**3.6**	**-25.9**	**-34.2**
**European zone**					
*Ae*. *biuncialis*	5.6	127.3	167.8	-17.5	-23.3
*Ae*. *cylindrica*	20.1	67.8	71.0	-30.9	-32.1
*Ae*. *geniculata*	17.6	13.7	8.0	-9.2	-18.3
*Ae*. *neglecta*	11.5	33.7	27.9	-12.7	-23.0
*Ae*. *triuncialis*	13.8	44.7	56.9	-7.7	-12.3
*Ae*. *ventricosa*	8.8	-56.6	-69.6	-57.2	-70.1
Richness (x100)	**77.5**	**36.4**	**38.7**	**-21.2**	**-27.8**
**Non-European zone**					
*Ae*. *biuncialis*	15.8	-7.8	-18.6	-29.4	-40.5
*Ae*. *cylindrica*	27.1	-16.6	-27.9	-30.5	-41.3
*Ae*. *geniculata*	7.8	-28.7	-39.6	-39.3	-53.0
*Ae*. *neglecta*	5.6	0.5	-6.7	-22.6	-30.9
*Ae*. *triuncialis*	23	-9.9	-14.1	-21.0	-25.6
*Ae*. *ventricosa*	4.1	-65.6	-83.3	-66.6	-84.0
Richness (x100)	**83.4**	**-15.5**	**-24.7**	**-29.8**	**-39.3**

Filling (%) corresponds to the number of suitable cells divided by the total number of cells (x 100). Column headings: C, 4.5 and 8.5 refer to the current, RCP4.5 and RCP8.5 projections, respectively; UM and NM refer to the universal and no migration hypotheses, respectively. Filling (%) corresponds to the percentage of cells predicted to be suitable. Change (%) expresses the rate of increase/decrease in predicted area of occupancy (PAO) relative to the current projection. The mean richness per cell was multiplied by 100. The change (%) in mean richness per pixel x 100 corresponds to the total change in PAO.

**Table 3:** Mean coordinates and predicted shifts.

	Mean		Shift							
			*UM*				*NM*			
	C		4.5		8.5		4.5		8.5	
Full grid	x	y	x	y	x	y	x	y	x	y
*Ae*. *biuncialis*	39.11	39.05	0.24	2.49	-0.74	3.34	-0.02	0.61	-0.97	0.85
*Ae*. *cylindrica*	43.55	44.37	-2.74	1.79	-3.38	2.02	-0.73	0.23	-1.67	0.38
*Ae*. *geniculata*	6.03	40.17	0.11	1.66	0.23	2.19	-0.86	0.90	-1.33	1.39
*Ae*. *neglecta*	12.19	39.64	0.29	1.61	0.63	1.87	0.83	0.38	1.49	0.55
*Ae*. *triuncialis*	28.61	38.75	-2.44	1.47	-2.09	1.93	-1.45	0.43	-1.33	0.52
*Ae*. *ventricosa*	0.38	39.90	-1.93	-0.56	-2.51	-0.05	-2.00	-0.53	-2.57	-0.03
**European zone**										
*Ae*. *biuncialis*	23.35	41.90	7.63	3.14	8.64	3.84	1.85	0.24	2.39	0.43
*Ae*. *cylindrica*	26.10	48.63	6.09	0.42	6.63	0.32	1.21	-0.07	1.32	-0.24
*Ae*. *geniculata*	2.78	42.55	0.12	1.02	0.16	1.39	-0.63	0.25	-1.02	0.55
*Ae*. *neglecta*	3.94	40.76	0.51	1.71	0.81	1.98	0.47	0.19	0.78	0.37
*Ae*. *triuncialis*	6.26	40.88	2.35	1.92	4.74	2.46	-0.52	0.11	-0.59	0.17
*Ae*. *ventricosa*	-0.86	42.81	-1.92	-1.38	-2.19	-1.33	-1.96	-1.39	-2.27	-1.35
**Non-European zone**										
*Ae*. *biuncialis*	44.02	38.17	1.63	0.71	0.74	0.82	0.12	0.59	-0.90	0.76
*Ae*. *cylindrica*	54.75	41.62	-2.71	0.77	-3.17	0.84	-1.92	0.43	-2.77	0.41
*Ae*. *geniculata*	12.45	35.47	3.86	0.86	5.51	1.34	1.61	0.50	2.29	0.80
*Ae*. *neglecta*	26.84	37.65	4.61	0.71	5.60	0.86	3.43	0.50	4.55	0.67
*Ae*. *triuncialis*	40.20	37.64	0.53	0.41	0.96	0.51	-0.08	0.43	0.28	0.53
*Ae*. *ventricosa*	2.70	34.45	-1.33	-0.05	-1.68	-0.18	-1.42	-0.02	-1.62	-0.15

Means were calculated over the cells predicted to be suitable under the current climate, with x and y standing for longitude and latitude in decimal degrees, respectively. Shifts were expressed by subtracting the 2050 climate coordinates from the current ones. Column headings: C, 4.5 and 8.5 refer to the current, RCP4.5 and RCP8.5 projections; UM and NM to the universal and no migration hypotheses, respectively.

**Table 4:** Prediction variability.

Species	Current (%)	RCP_4.5_ (%)	RCP_8.5_ (%)
*Ae*. *biuncialis*	7	9	10
*Ae*. *cylindrica*	18	15	13
*Ae*. *geniculata*	4	5	5
*Ae*. *neglecta*	3	4	4
*Ae*. *triuncialis*	6	6	7
*Ae*. *ventricosa*	7	4	4

Difference in filling estimate (%) between the maximum and minimum statistical projection grids (universal migration hypothesis) provided by MaxEnt.

**Fig 2:**
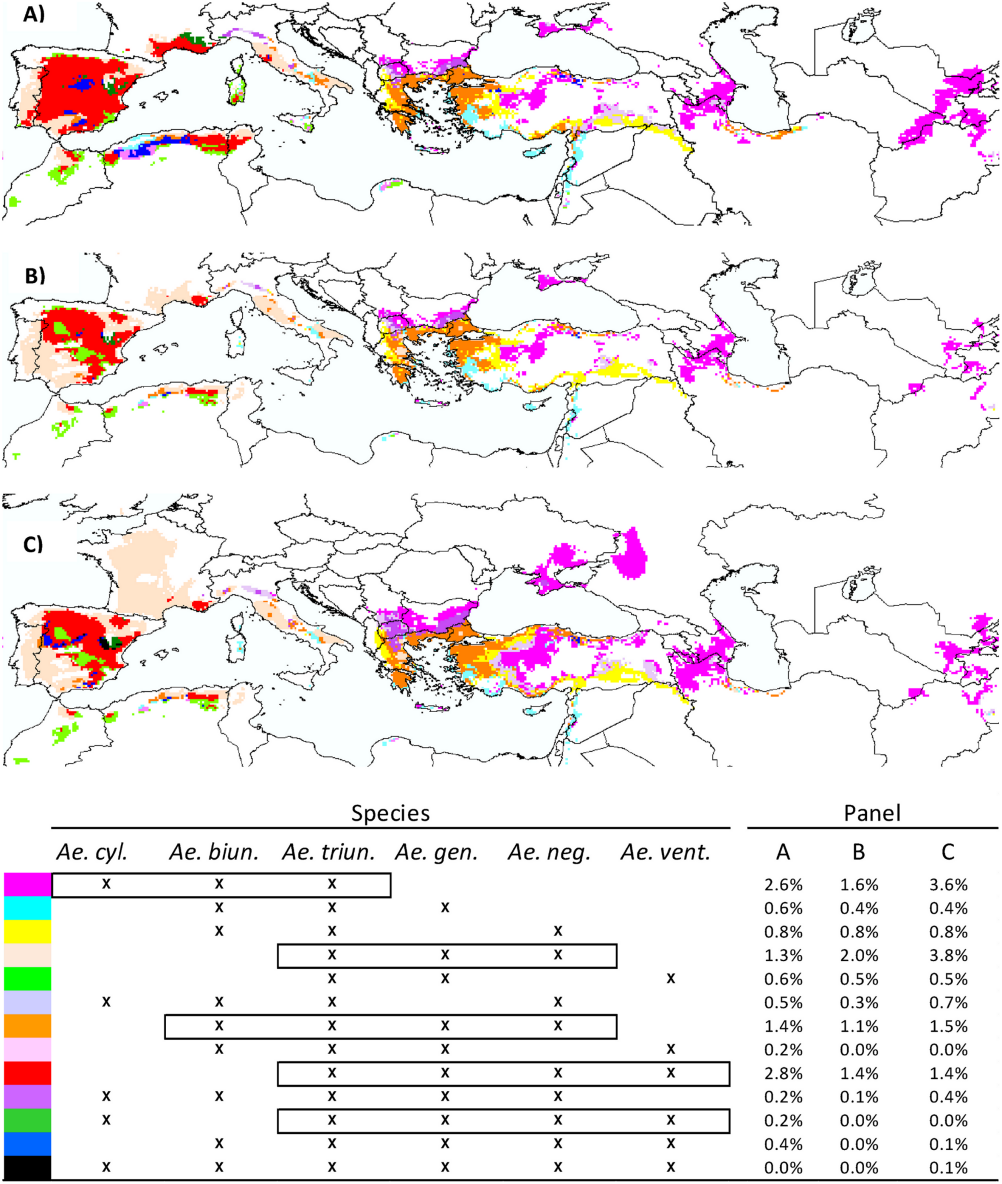
Most frequent potential associations of at least three species: RCP_4.5_. Predicted associations for **(A)** the current climate, **(B)** RCP_4.5_ under the no migration hypothesis and **(C)** RCP_4.5_ under the universal migration hypothesis. All classes of potential species richness ranging from three to six are represented. The color code is given below. The frequency of each association is also reported (in % of the total number of grid cells). Boxes indicate the most frequent associations in all RCP-by-migration hypothesis combinations.

More than 60% of the cells predicted to be concurrently suitable for at least four species were located in southern Europe (Fig 1). Such predicted diversity hotspots were mapped throughout Spain, eastern Portugal, southern France, Italy and part of the Balkan Peninsula in Europe, whereas outside Europe they were more restricted to coastal regions of Algeria and border areas of Turkey (Fig 1). As a whole, Turkey appeared nonetheless as a major potential diversity spot, as all six species—but *Ae*. *ventricosa*—were reported and predicted to benefit from suitable conditions in this country (Fig 1). Among the cells predicted to be concurrently suitable for at least three species (12% of grid cells), some species associations were more frequent than others and were geographically structured (Fig 2). The most frequent of such associations involved a triplet consisting of *Ae*. *triuncialis*, *Ae*. *geniculata* and *Ae*. *neglecta* or quadruplets with *Ae*. *ventricosa* or *Ae*. *biuncialis* as additional species, from westward to more southern central regions (Fig 2). The second most frequent association noted from southern central to eastward regions involved the triplet *Ae*. *triuncialis*, *Ae*. *cylindrica* and *Ae*. *biuncialis* (Fig 2).

### 2050 projections

In the following, RCPs were averaged unless otherwise stated. Under the universal migration hypothesis, the predicted richness (Fig 1 and S3 Fig) was predicted to increase from 0.81 to 0.85 and to decrease to 0.56 species per cell, if no migration could occur (Table 2). Suitable conditions for all species but *Ae*. *ventricosa* were predicted to shift northward, with shifts ranging from approximately 1.5° to more than 3° in latitude across the full extent of the projection grid (Table 3). If no migration could occur, smaller northward shifts were also predicted, indicating that the sites predicted to remain suitable by 2050 were distributed more northward, on average (Table 3 and S5 Fig). Overall, RCP_4.5_ somewhat appeared to be the most favorable/least unfavorable RCP for all six species. For all species, the net gains (gains minus losses) were nearly equal or larger under RCP_4.5_ than under RCP_8.5_ and losses were systematically smaller under the former than under the latter RCP (Table 2).

Under the universal migration hypothesis and for both RCPs, *Ae*. *biuncialis*, *Ae*. *neglecta*, *Ae*. *cylindrica* and *Ae*. *triuncialis* were predicted to benefit from high to moderate increases in PAO (+25%, +19%, +14% and +10%, respectively). Conversely, losses exceeded gains for both *Ae*. *ventricosa* and *Ae*. *geniculata*, resulting in a decrease in their projected PAO. However, whereas this reduction was moderate for *Ae*. *geniculata* (-1%: RCP_4.5_; -8%, RCP_8.5_), it turned out to be quite substantial for *Ae*. *ventricosa*, with nearly no differences between migration hypotheses (-60%, RCP_4.5_; -74%, RCP_8.5_). Under the no migration hypothesis and excluding *Ae*. *ventricosa*, the greatest predicted reductions in PAO were for *Ae*. *cylindrica* and *Ae*. *biuncialis*, under both RCPs (-34% and -32%, respectively). The predicted reduction for *Ae*. *geniculata*, *Ae*. *neglecta* and *Ae*. *triuncialis* ranged from -25% to -19%, respectively. Despite these differences, the species ranking order for filling estimates did not change much relative to current estimates (S3 Table).

The highest contrast was found between the two main geographical areas. These areas differed markedly with respect to cell gains and losses predicted by 2050. Under both RCPs, 64% of total cell losses (i.e. summed over species) were located outside Europe, whereas 78% of the gains were located within Europe. This was a general trend as the potential net gains were positive within the European zone for all species but *Ae*. *ventricosa* and quasi-null or negative outside Europe under both RCPs (Table 2). Overall, the models therefore predicted a potential decline in suitable conditions for *Aegilops* species outside the European zone (-20%) and a parallel increase in Europe (+38%). If no migration could occur, the decline was predicted to be more acute outside (-35%) than within the European zone (-24%, details in Table 2).

In all cases, Europe was predicted to be hosting more than 60% of cells concurrently suitable for at least four species by 2050. However, the number of such sites decreased not only outside but also within the European zone, even under the universal migration hypothesis (Figs 1 and S3). This finding was mostly attributable to the predicted reduction in PAO of *Ae*. *ventricosa* in the southwestern Mediterranean area and to a lesser extent of *Ae*. *neglecta* and *Ae*. *geniculata* in Spain (S4 Fig). The southwestern Mediterranean area appeared to be one of the regions where the species association pattern changed the most in all RCP-by-migration hypothesis combinations (Figs 2 and S4). Eastward, numerous sites concurrently suitable for *Ae*. *triuncialis*, *Ae*. *cylindrica* and *Ae*. *biuncialis* were predicted to be lost. Alongside these losses, however, conditions suitable for this common triplet were also predicted to expand around the Azov Sea, but also towards the north of Bulgaria and the center of Turkey. Westward, a similar expansion was predicted in France for the triplet comprising *Ae*. *triuncialis*, *Ae*. *geniculata* and *Ae*. *neglecta*.

### Current potential sympatry

**Fig 3:**
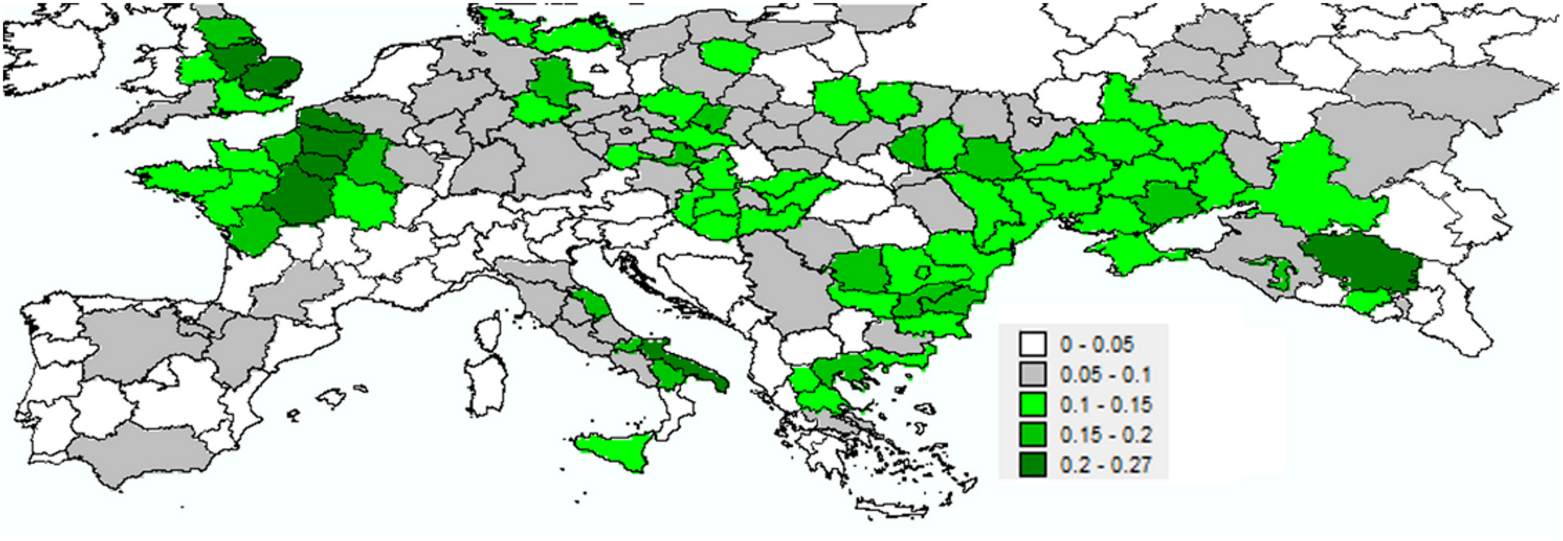
Average wheat harvested area according to various extents of territorial units. Data were divided by the total area of each territorial unit. Averages were calculated within the 2000–2009 time range, with variations depending on data availability. Cell values included in each unit served as a constant proxy over time for the cultivated wheat encounter probability.

**Fig 4:**
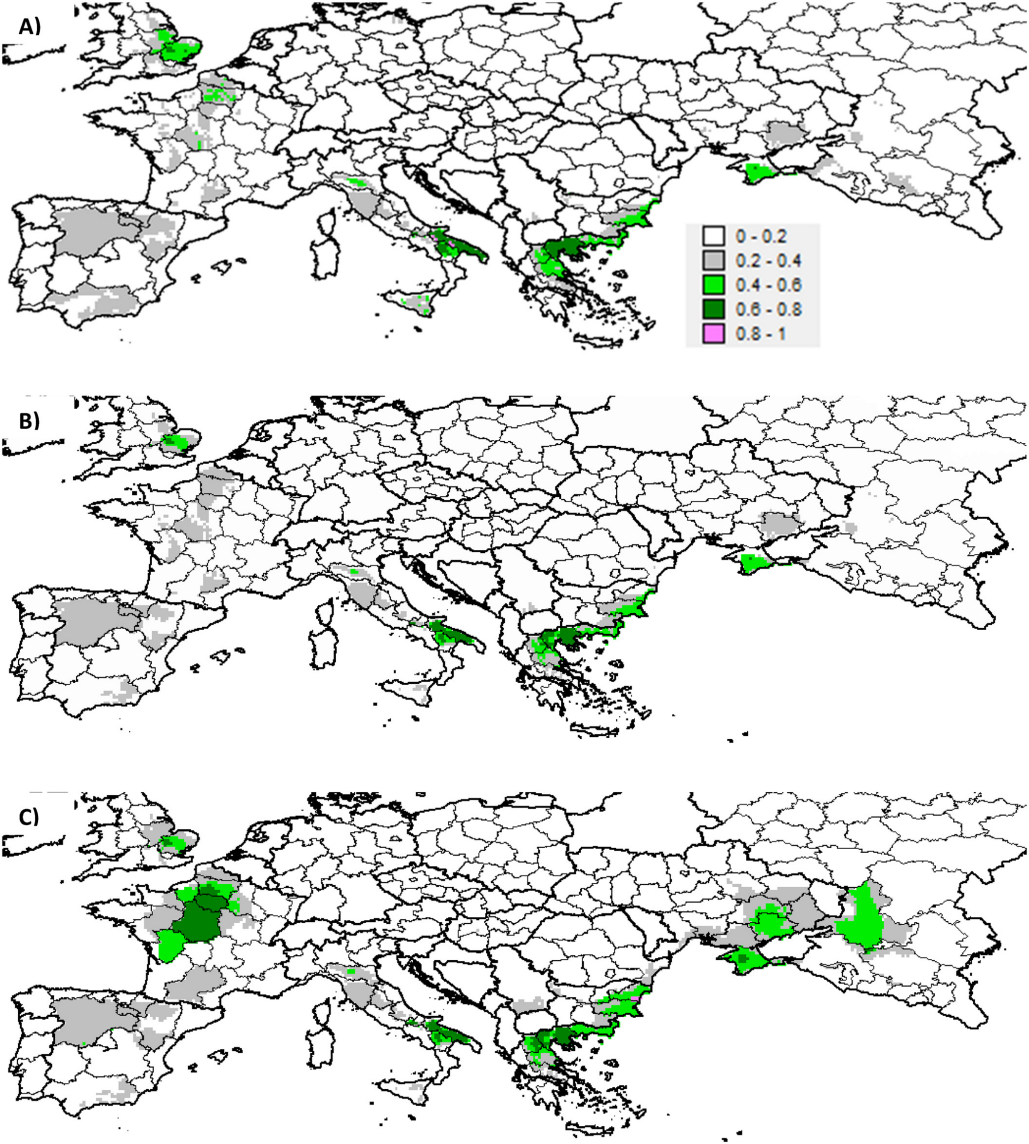
Global potential sympatry index between the six *Aegilops* species and cultivated wheat in the European zone: RCP_8.5_. Global sympatry index (richness x wheat proxy) for **(A)** the current climate, **(B)** RCP_8.5_ under the no migration hypothesis and **(C)** RCP_8.5_ under the universal migration hypothesis.

Species associated with the largest PAOs in the European zone were also associated with the largest PSIs (Tables 2 and 5, S6 and S7 Figs). However, the highest mean PSI was obtained for *Ae*. *biuncialis* (0.09), the species characterized as having the smallest current PAO in Europe (Tables 2 and 6). At the country level, despite the high predicted species richness (Fig 1), Portugal was nonetheless characterized by a low PSI, due to low probabilities of encounters with cultivated wheat across its territorial units (Figs 3 and 4). The reverse situation was noted for the United Kingdom (excluding Scotland). Spain, France and Ukraine scored the highest current PSIs in Europe (Table 7). However, mean PSIs were highest for countries comprising the southernmost territorial units (Table 7, Fig 4).

**Table 5:** Species PSI and predicted changes.

	PSI	Change (%)			
		*UM*		*NM*	
Species	C	4.5	8.5	4.5	8.5
*Ae*. *biuncialis*	127.1	148.9	187.4	-17.5	-22.6
*Ae*. *cylindrica*	419.8	46.8	50.8	-31.7	-31.6
*Ae*. *geniculata*	341.9	21.1	14.8	-9.0	-17.5
*Ae*. *neglecta*	167.7	65.0	56.1	-11.4	-22.4
*Ae*. *triuncialis*	208.4	92.7	121.0	-7.2	-10.6
*Ae*. *ventricosa*	156.3	-63.4	-73.5	-63.9	-73.9
Total	1421.1	46.5	51.6	-22.5	-27.9

Change (%) expresses de rate of increase or decrease in PSI relative to current projections. Column headings: C, 4.5 and 8.5 refer to the current, RCP4.5 and RCP8.5 projections; UM and NM to the universal and no migration hypotheses, respectively.

**Table 6:** Mean PSIs and predicted changes.

	Mean PSI	M_G_/M_L_		Change (%)			
				*UM*		*NM*	
Species	C	4.5	8.5	4.5	8.5	4.5	8.5
*Ae*. *biuncialis*	0.088	1.15	1.13	9.5	7.3	0.0	1.0
*Ae*. *cylindrica*	0.081	0.78	0.81	-12.5	-11.8	-1.1	0.8
*Ae*. *geniculata*	0.076	1.34	1.28	6.5	6.3	0.2	1.0
*Ae*. *neglecta*	0.057	1.84	1.58	23.4	22.1	1.5	0.8
*Ae*. *triuncialis*	0.059	2.03	2.21	33.1	40.8	0.5	1.9
*Ae*. *ventricosa*	0.069	0.65	0.63	-15.7	-13.0	-15.5	-12.6
Total	0.071	1.13	1.19	7.4	9.3	-1.7	-0.1

Species means were calculated as PSI divided by the number of suitable cells (conditional mean). Total corresponds to total PSI (i.e. summed over species) divided by the number of suitable cells summed over species. The M_G_/M_L_ ratio corresponds to the mean PSI computed over gained sites divided by the mean PSI over lost sites. Change (%) expresses the rate of increase or decrease in mean PSI relative to current projections. Column headings C, 4.5 and 8.5 refer to the current, RCP4.5 and RCP8.5 projections; UM and NM to the universal and no migration hypotheses, respectively.

**Table 7:** Country PSI (top eight).

	PSI	Mean PSI	Change (%)			
			*UM*		*NM*	
Country	C		4.5	8.5	4.5	8.5
Bulgaria	82.7	0.19	27.4	44.4	-13.5	-7.9
France	185.3	0.08	139.9	145.1	-15.3	-17.0
Greece	123.4	0.25	-1.1	-11.7	-8.5	-19.0
Italy	174.5	0.15	-14.5	-32.4	-28.8	-43.8
Russia	76.6	0.01	396.4	473.8	-27.3	-19.8
Spain	302.1	0.16	-8.6	-16.3	-14.4	-23.2
UK	98.2	0.13	-19.0	-30.1	-43.8	-51.6
Ukraine	183.4	0.07	91.5	125.9	-9.7	-8.3

Country PSI was calculated as the sum of PSI over species. Mean PSI corresponds to PSI divided by the total number of pixels included in each country. Change (%) expresses the rate of increase or decrease relative to current projections. Column headings C, 4.5 and 8.5 refer to the current, RCP4.5 and RCP8.5 projections; UM and NM to the universal and no migration hypotheses, respectively.

### 2050 potential sympatry

As the models predicted an increased potential *Aegilops* species richness in Europe by 2050 (+38%), a rise in European PSI was expected under the universal migration hypothesis (Table 5, Fig 4, S8 Fig). Averaging over RCPs yielded a 49% increase in European PSI. However, the changes in species mean PSIs were less foreseeable. The estimated change for the global mean across species was moderate (+8%), but ranged from -12% and -15% for *Ae*. *cylindrica* and *Ae*. *ventricosa* to up to +37% for *Ae*. *triuncialis*. On average, the gained sites were characterized by a higher probability of encountering wheat than the currently suitable sites, for four of the six *Aegilops* species, while the reverse trend was noted for sites predicted to be lost (Table 6).

The countries encompassing the largest predicted net gains in suitable cells contributed the most to the increase in European PSI, i.e. Russia, France and Ukraine. Under both RCPs, the 2050 PSI was predicted to become two-fold higher than the current index in Ukraine, more than two-fold in France and five-fold in Russia (Table 7). In other countries (top eight PSIs), apart from Bulgaria, the country PSI was predicted to decrease by 2050. In France, the species contributing the most to the potential increase were *Ae*. *triuncialis* and *Ae*. *neglecta*, whereas *Ae*. *cylindrica* and *Ae*. *biuncialis* were predicted to be the main contributors in Ukraine and Russia. If no migration could occur (Fig 4 and S8 Fig), the rate of decrease in European PSI was nearly the same as that of the European species richness (~-25%, Tables 2 and 5). The predicted decrease in PSI for individual species was also in the same order of magnitude as the rate of decrease in PAO (Tables 2 and 5). At the country level, despite substantial richness loss, particularly in Spain, Italy, France and Greece, the ranking order for country PSI and mean PSI did not change much relative to the current order (Table 7).

## Discussion

To our knowledge, this study provided the first broad scale evaluation of both current and future potential sympatry levels between crop wild relatives and a crop species. In the process, the models provided proxies to current geographical distributions, diversity structuration and hotspots that are fairly consistent with present knowledge on the six most common *Aegilops* species in Europe [7]. The global results derived from the 2050 projections are also consistent with the growing body of ENM and ecological literature reporting either predicted or already achieved northward shifts in numerous plant and animal species and/or increased extinction risks as a result of ongoing climate change [20–23, 26]. However, the proposed inferences are inevitably dependent on the samples, methods and scenarios used. Nevertheless, these projections offer grounds for questioning the impact of climate change on wheat to *Aegilops* gene flow and conservation issues regarding these wild relatives. Several aspects will now be addressed, primarily focusing on trends and limitations, while proposing potential improvements.

### Variable importance

With respect to the typical life cycle of these annual plants, some climatic variables were expected to be important across species in delineating suitable areas (S2 Appendix). In the Mediterranean Basin, the studied species were described as winter annuals, germinating after a hot/dry summer following the onset of autumn rainfall. If rainfall and temperature keep on being favorable, winter growth could be continuous with plants then flowering during the subsequent spring and fruiting from early to late summer. Once shattered, seeds within spikelets enter a variable phase of dormancy, thus preventing germination while unfavorable hot and especially dry conditions still prevail [51–54]. Across the selected models, the most recurrent contributing temperature variables were associated with the coldest periods (S2 Appendix). On a large scale, minimum temperatures are expected to be particularly important in limiting the poleward range limit of plant species [55]. This finding was also consistent with the biological features of *Aegilops* species, which typically overwinter at the vegetative stage. Regarding deviations between the training sample means and background means, the species ranges appeared to be generally limited northward by the requirement of relatively mild winter temperatures, with one exception for *Ae*. *cylindrica*. With regard to temperature during the coldest periods, *Ae*. *cylindrica* was the only species presenting lower sample mean values than the average across its background (Fig A2S1 in S2 Appendix). It was also characterized by lower sample means than all the other species (Table A2S1 in S2 Appendix). In the *Aegilops* genus, D-genome bearing species are generally found at higher latitudes and elevations [7]. Accordingly, cold-resistance was suggested to explain this pattern [56]. However, while cold-resistance genes were found in the diploid parental species *Ae*. *tauschii* (DD) [57], *Ae*. *ventricosa* (DDNN) did not appear to be particularly cold-resistant (S2 Appendix), leaving little room for a simple D-genome bearing explanation of cold tolerance. Yet the D-genome of *Ae*. *cylindrica* was previously shown to present small rearrangements compared to the diploid parental species *Ae*. *tauschii*, in contrast with *Ae*. *ventricosa* [58]. In any case, *Ae*. *cylindrica* was experimentally shown to be resistant to heavy frost at the vegetative stage, surviving better than *Ae*. *geniculata* and *Ae*. *neglecta* under the same conditions [56]. Importantly, *Ae*. *cylindrica* could also avoid winter harshness as it was reported to be able to germinate and flower in spring of the same year if sufficient vernalization is achieved [59]. Exposure to chilling temperatures (vernalization) at early life stages is known to accelerate/enhance the later growth of reproductive structures (flower and tiller number). Therefore, it can be hypothesized that this variable is also involved in limiting the population growth rate at southward edges, as the mean temperature becomes increasingly warm southwards during winter. However, it seems more likely that the southernmost limits were mainly due to the drop in annual rainfall below the minimal water requirements of these species. In turn, the most recurrent contributing precipitation variables were those associated with the warmest/driest periods (S2 Appendix). The importance of these variables may be interpreted in various ways since complex relationships among predictors were allowed to be fitted. Overall, however, species means for the warmest quarter were all below the background means, which is consistent with the generally acknowledged importance of the difference in precipitation between wet and hot-dry seasons with regard to *Aegilops* adaptation and biology.

### Current projections

With respect to the reference distributions that were based on herbarium data and published by M. van Slageren (hereafter RDs, see [7]), the quality of our occurrence samples was satisfactory overall, partly due to their non-independence. When cross-checking M. van Slageren’s sources against ours (accessed from Gbif), we found that several herbarium collections overlapped (BC, G, L, LD, MO; S1 Table). However, some regions were poorly represented in our samples compared to RDs. Imbalanced sampling probably affected the models when estimating the probability density function of climatic variables associated with the sampling points [36]. Nevertheless, the models often succeeded in identifying undersampled regions as suitable. Italy is a particularly relevant case. In this country, all six species were reported in RDs (see also [60]). Although only *Ae*. *geniculata* was relatively well represented in Italy in our sample, all of the species models identified some Italian regions as suitable. To some degree, this could be regarded as indicative of good model behavior. Conversely, however, the extent of the adverse effect of the sampling imbalance was not investigated.

When comparing RDs [7] with the projected current distributions, binary vs. continuous inferences were found to have their own respective qualities and flaws. Although they flattened the continuous suitability index, binary maps were found to be advantageous in properly highlighting the historical and actual areas where the species were reported, whether as native/naturalized or adventive plants. Continuous results, on the other hand, adequately designated hot spot regions, but sometimes imperfectly reflected the known relative abundance of particular species across regions. This could be due to a number of causes. For instance, as already suggested by a number of authors, ENM suitability can be conceptually considered as a surrogate for maximum potential attainable densities. Suitable localities may have not been fully occupied by a species for reasons related to its particular migration history. Alternatively, however, if factors driving abundance are not included in the model, high abundances may be found in highly suitable places, but low abundances can be found in both highly and marginally suitable places [61]. Such missing factors could be edaphic or edaphic-correlated. Several authors have previously reported/suggested soil type affinities in *Aegilops* species (*e*.*g*. [7, 62]; but also see [63]). Yet, a recent study showed that adding edaphic variables to climatic ENMs globally increased the AUC metric of accuracy of various plant predictive models [64]. When removed, the models were interpreted as picking up climatic constraints on distributions, while making false positive predictions within the climatically suitable areas [64]. Overall, we feel that there are reasonable grounds to consider that the selected climate models captured the climatic tolerances of these *Aegilops* species, but yielded optimistic current suitability (thus future suitability as well), especially when projected to northernmost areas (see [7]). Therefore, additional caution should be taken when considering the projected suitability in these areas. This is particularly relevant for *Ae*. *cylindrica* as this species was associated with the highest prediction variability.

### 2050 projections

Another possible drawback might be related to the change in correlation matrix of the Bioclim variables between background vs. current and vs. 2050 projection grids (not shown). As stated by Elith et al. [47], such changes could be particularly problematic if predictors are only indirectly related to the species distribution (distal rather than proximal variables). In such cases: ‘*the selected set might together represent the unmeasured directly influential variable reasonably well*, *but if correlations between them change*, *prediction will be compromised’*. We observed (not shown) that the pairwise correlation matrices were moderately dissimilar between the background and current projection grid, highly similar between both RCPs, which differed markedly from the current projection matrix. In *Aegilops* species, one important candidate proximal variable in shaping their distribution is competition with other plants, especially perennial herbs that thrive better in nutrient and water-rich conditions (pers. obs.; [7, 60]). There are complex feedback relationships between plant community compositions, soil characteristics and climate [64]. However, the associated climate variables might well reflect the influence of soil and competition, but as the correlations between climate variables will (likely) change in the future, the correlation between competition/soil and climate variables might change as well.

### Migration hypothesis

For most of the species we focused on here, tentative conclusions that could be drawn differ considerably depending upon which migration hypothesis is considered. However, a more realistic assumption would lie somewhere in between these two extremes [26]. Morphological features of *Aegilops* species—awns and awn hairs on disarticulating spikes/spikelets—ensure efficient epizoochorous seed dispersal. Based on the high germination rate of *Ae*. *cylindrica* seeds recovered from cattle feces [65], it was previously proposed that herbivores may also be an important endozoochorous dispersal vector for the genus [10]. However, the lower palatability of *Aegilops* possessing very long and dense awns, such as *Ae*. *triuncialis* [66], suggests that the importance of this mechanism may vary depending on the species. Similarily, the relative efficiency of the epizoochorous dispersal mechanism may well depend on the awn length/density. Other dispersal mechanisms are also involved, including wind [67] and water (spikes/spikelets float: [68–69]). However, long distance dispersal—particularly across fragmented habitats and beyond natural barriers—may be required to keep pace with climate change [70]. It has been previously suggested that human-mediated transportation, mostly in open disturbed areas such as pastures, roads and field margins, might have contributed to the expansion of these colonizing species [71]. A phylogeographical study focused on *Ae*. *geniculata* recently highlighted that this species likely occupied southern Europe before the Last Glacial Maximum, i.e. before the spread of agriculture [72]. However, this study [72] also revealed more recent migration and numerous intra-specific introgression events from northern Africa to southern Europe, consistent with long-distance dispersal patterns following human trade routes. *Ae*. *cylindrica* and *Ae*. *triuncialis* introductions and spread were even more recent, in addition to *Ae*. *geniculata* in USA (http://www.plants.usda.gov/core/profile?symbol=aege). Long-distance dispersal has so far mostly if not exclusively been shown to be human-mediated, but this pattern nevertheless suggests that these species may be able to migrate fast enough for their range limits to track climate change patterns.

### Establishment of new range limits

The success of bioclimate reconstruction models in simulating Holocene distribution changes for some species highlights the efficiency of the Climatic Niche Modeling approach in predicting future continental-scale plant species distribution patterns [24, 73]. However, it was also appropriately emphasized that population genetics theory predicts that the establishment of new ranges under the pressure climate change involves more than migration. Indeed, dispersal is likely to be random with regard to adaptation to the conditions where a seed lands. Random, selective, recombination and demographic events are expected to interact with migration throughout any range shift [74]. Therefore, the success in establishing new ranges likely depends on the adaptive potential of the shifting species. It has long been noted that, unlike their diploid parents, most allotetraploid *Aegilops* species are present simultaneously in different ecozones [75–76]. Phylogeographically, from somewhat diffuse southern-southwestern areas to Transcaucasia (putative region of origin of the genus; [7, 77]), the six allotetraploid *Aegilops* species focused on here spread much wider than any of their respective parental diploid species (except for the eastward spread of weedy *Ae*. *tauschii*; parental genome D; [7]). These species are generally considered as having great adaptive potential. As first stated by Zohary and Feldman [78] regarding allopolyploid *Aegilops*, outcrossing and inter-specific hybridization combined with predominant selfing constitute a very efficient genetic system in promoting rapid evolution. A recent study revealed evidence supporting the idea that increased genetic diversity via the number of recurrent polyploidization events is associated with increased ecological amplitude in these species [79]. As for other successful polyploids, the role of allopolyploidy *per se* [80] and the relative importance of environmental plasticity as opposed to strict local adaptation in explaining ecological amplitude and invasiveness are still open questions [10]. Surprisingly, there has been little work on the morphological and genetic variation of *Aegilops* populations related to their natural environment [76]. In a few studies, intra-specific life history traits were shown to vary across geographical/ecological zones at both large and smaller scales [10, 56, 76, 81]. However, the quantification of genetic components of phenotypic variation (genotype/population levels) in relation to ecological gradients has been complicated by the existence of complex maternal effects in *Aegilops* species, as also noted in other Poaceae species [82]. Seed weight and various correlated fitness traits in progeny were shown to depend on the spikelet position of the seeds on the mother plants and on the environmental conditions in which the mother plants were raised [51–54]. Ultimately, however, maternal effects, genetic variation and phenotypic plasticity were all suggested to contribute to the adaptive/colonizing potential of allopolyploid *Aegilops* species [10, 51, 83]. Yet, the rapid successful expansion of *Ae*. *triuncialis* and *Ae*. *cylindrica* in USA despite strong genetic bottlenecks is still challenging theoretical expectations [83–84], as also noted for other invasive species (but see [85]).

### Conservation

Crop wild relatives are invaluable genetic resources for crop improvement and the conservation of their biodiversity is a major issue addressed by different worldwide programs and organizations. As members of the secondary gene pool of cultivated wheat, *Aegilops* species have substantially contributed to genetic improvements in the past and are expected to further participate in broadening the genetic base of wheat cultivars [86–87]. It is therefore critical that *Aegilops* species are efficiently conserved *in situ* and *ex situ* so as to be available for use by breeders [87].

According to IUCN criteria [26, 88], a projected range loss of >50% and >30% classifies species as endangered and vulnerable, respectively. With regard to the climate scenarios we used, one of the most critical trends was the marked PAO reduction of *Ae*. *ventricosa*. Associated with a decrease consistently projected to be greater than 50% by 2050, *Ae*. *ventricosa* could become an endangered species. The status of the other concerned species is more debatable as only *Ae*. *biuncialis* and *Ae*. *cylindrica* were predicted to pass though the vulnerability threshold if no migration could occur.

It was recently estimated that most *Aegilops*-rich countries have likely been undersampled for *ex situ* conservation purposes and that additional diversity could potentially be found. Apart from *Ae*. *cylindrica*, all of our studied species were recently given an *ex situ* conservation priority (although generally low) in several countries. With respect to the prioritized species included here, the coastal regions of North Africa, western Spain, southern Greece and Italy were among the most frequently proposed regions for further conservation sampling [87]. In the present study, these regions were all predicted to undergo substantial richness loss by 2050, thus providing support for the *ex situ* conservation priorities recently proposed. In line with our results, the only species actually associated with a higher priority level is *Ae*. *ventricosa* (medium level). Still, the conservation priority level of this species might be insufficient gauged, especially when considering that the presence of a D genome eases the transfer of genes of interest to bread wheat as compared to other *Aegilops* species. With respect to the above geographical sampling prioritization, northern Africa should be the focus of rapid sample collection for two main reasons. Genetic diversity has been previously reported higher on the southern coast than on the northern coast of the Mediterranean Sea, in *Ae*. *geniculata* [72]. This might be the case for other species found in northern Africa. As the Mediterranean Sea is an important geographical barrier, escaping from climate change via human-mediated overseas migration would likely result in major genetic diversity loss.

From the *in situ* conservation perspective, Maxted et al. [87] only identified one worldwide genetic reserve devoted specifically for conserving *Aegilops* species. Based on current spatial analysis of species richness (N = 22 species), five complementary areas for new genetic reserves have been proposed [87]. Our results raise concerns about most of these sites as three of these six areas were predicted to undergo a decrease in suitability by 2050. Moreover, none of these areas intersect large zones where suitable conditions for *Ae*. *ventricosa* were predicted to remain, such as in Spain. Overall, this stresses the need for more active demographic and genetic monitoring of *Aegilops* populations than is actually carried out, as climatic conditions are changing. Hence, efforts should be made to implement and update consensus projections using different inferential methods/models for these crop wild relatives.

### Projected sympatry levels

For most of the considered species projected to be present in Europe by 2050, the absolute change in PAO was greater under universal migration than under the no migration hypothesis. PSI reflected the same trend as wheat is cultivated throughout Europe. However, the projected increase in European PSI was larger than for PAO, whereas both estimates decreased at similar rates under the no migration hypothesis. This indicated that, on average for most species, sites predicted to become suitable in the future currently encompass a greater wheat surface area than the sites predicted to be lost by 2050. At the country level, potential colonization sites were mainly located in three countries: Russia, France and Ukraine. These countries are among the top four wheat producers in Europe, and they are also the top three countries with respect to the wheat harvested area (http://www.faostat3.fao.org/). These countries were here predicted to become the most exposed to increased sympatry levels. This may have major consequences on the rate of wheat-to-*Aegilops* gene flow which could in turn result in increased weediness and/or decreased adaptation to natural ecosystems. However, whether a rise in wheat-to-*Aegilops* hybridization potential will occur or not in the future depends upon many unknown parameters. It is currently known that all six species hybridize with bread wheat to some extent [7–8]. Although predominantly selfing species, F1 hybrids can constitute as much as 7–8% of seeds collected from *Ae*. *cylindrica* individuals ([13, 89]. High fertility restoration in backcross lines is also possible and could occur over only a few generations. However, all of these aspects were well studied only in the D genome bearing *Ae*. *cylindrica* and essentially in USA (e.g. [4]). Self-fertility nevertheless sometimes directly occurs in F1 hybrids involving other *Aegilops* species via the production of unreduced gametes and amphiploid offspring [90–92]. However, mating systems and fitness and thus actual hybridization and migration could also be affected by climate change. Specifically, outcrossing and interspecific hybridization combined with predominant selfing could change in relative importance in the future. In wheat, as in *Aegilops* species, wider and longer flower opening than commonly observed has been reported in some instances. This was interpreted as resulting from low male fertility, leading in turn to increased opportunity for cross-pollination [93–94]. In a number of species, including wheat, reduced male fertility/pollen viability can be induced by hot temperature at the microsporogenesis and anthesis stages [95–97]. Therefore, and as already observed in wheat (e.g. [98]), stress resulting from higher to occasionally much higher than optimal spring temperatures might reduce seed set in *Aegilops* species as well. Concurrently, however, transient pollen damaging temperatures might also lead to higher outcrossing and hybridization rates [93]. This outcome can be hypothesized based on several observations. Female gametophytes are less sensitive to heat stress than pollen at the anthesis stage in cereals [99]. In addition, a proportion of anthers could avoid transient stress as flowers regularly enter anthesis on different days, either within a single genotype/species [100] or between species via overlapping flowering windows (e.g. [8]). Reduced seed set and thus propagule pressure could lower the colonizing capacity of *Aegilops* species. However, increased outcrossing rates may in turn result in greater genetic variation for all traits, including adaptations to the specific conditions that prevail where the seeds land [101]. Still, wheat-to-*Aegilops* hybridization rates might also increase following the development/comeback of hybrid wheat, as several seed manufacturers intend to release new such varieties in the near future (e.g. for 2020 see [102]). If enhanced male fertility in paternal lines is selected by breeders for hybrid seed production [103], a heavier pollen load and thus greater hybridization opportunities could arise from the spread of such hybrid-wheat cultivars.

In this study, it was also assumed that no major change in European wheat harvested areas would occur by 2050. However, the stability of the wheat map in Europe over the next 40 years is debatable (e.g. see [104]). Since anthropogenic land-use and land-cover change (LULCC) affects climate through both emissions and albedo, projected gridded trajectories of LULCC represent a new mandatory dataset for the four selected IPCC5 Integrated Assessment Modeling (IAM) teams (RCP modelers). However, these projections are associated with a great level of uncertainty [105]. Indeed, various scenarios with very different regional changes in LULCC and industrial activity may be consistent with the very same RCP [101, 102], and cropland use projections are very sensitive to assumptions such as increases in crop yield, changes in diet, or how agricultural technology and intensification is applied [44, 106–107]. At present, choosing which IAM model/projection to use is unfortunately inseparable from the choice of scenario [108]. Land-use modeling is a challenging and active area of multidisciplinary research. As agricultural land-use models can be very complex and time consuming, a promising meta-modeling avenue was recently proposed, promoting parameter flexibility and decreased running time [109]. Ultimately, however, crop-specific gridded projections resulting from different socioeconomic and crop simulation models should be more available. This would allow for more comprehensive investigations regarding many issues in the biological and ecological fields, including those arising from the sympatry between cultivated species and their wild relatives.

## Conclusion & Perspectives

Environmental niche modeling using only presence data has limitations precluding satisfactory assessment of the predictive model quality (e.g. [110]). Nevertheless, and paraphrasing state-of-the-art researchers in the field, ENM allows studies on biological issues that are impossible to address using other methods. In turn, however, ‘*the inferences made from the models are only as good as the models themselves*’ [46]. As such, this work represents one small step in a more general reflection about possible changes in crop-to-wild relative gene flow under the pressure of climate change, but also of increasing crop demand as a result of the growing global population [111]. In Europe, legal issues towards transgenic crop species are very sensitive matters. Although no transgenic wheat variety has yet been released anywhere in the world, it is likely pending (e.g. see: http://isaaa.org/resources/publications/pocketk/38/default.asp). The European Commission has financed important research programs to assess risks and impacts of transgenic crop species, e.g. SIGMEA (gene flow oriented). However, SIGMEA focused essentially on maize and oilseed rape. As stated in the SIGMEA conclusions [112]: ‘*The knowledge base for wheat and rice in Europe is much less than for others crops…Further research is needed on cross pollination and the life cycle of these species and their wild relatives in Europe’*. Clearly, further investigations involving distribution modeling/monitoring of these species, seed migration, hybridization rates and fertility restoration in a range of environmental conditions are needed.

## Supporting Information

S1 Appendix Background grids. (PDF)

S2 Appendix Sample means and variable contribution. (PDF)

S1 Fig Potential predicted distributions: RCP_4.5_. (PDF)

S2 Fig Potential predicted distributions: RCP_8.5_. (PDF)

S3 Fig Potential species richness: RCP_8.5_. (PDF)

S4 Fig Most frequent potential associations of at least three species: RCP_8.5_. (PDF)

S5 Fig Summary of changes in potential area of occupancy. (PDF)

S6 Fig Potential sympatry index between cultivated wheat and individual *Aegilops* species in the European zone: RCP_4.5_. (PDF)

S7 Fig Potential sympatry index between cultivated wheat and individual *Aegilops* species in the European zone: RCP_8.5_. (PDF)

S8 Fig Global potential sympatry index between the six *Aegilops* species and cultivated wheat in the European zone: RCP_8.5_. (PDF)

S1 File Complete dataset. (XLSX)

S1 Table Institutions contributing to the dataset. (PDF)

S2 Table Names of the climate models. (PDF)

S3 Table Detailed filling estimates and species ranking order. (PDF)

## References

[1] Ellstrand NC, Prentice HC, Hancock JF. Gene flow and introgression from domesticated plants into their wild relatives. Ann Rev Ecol Syst. 1999;30:539–563.

[2] Haygood R, Ives AR, Andow DA. Consequences of recurrent gene flow from crops to wild relatives. Proc R Soc B. 2003;270:1879–1886. 14561300 10.1098/rspb.2003.2426 PMC1691463

[3] Jenczewski E, Ronfort J, Chèvre A-M. Crop-to-wild gene flow, introgression and possible fitness effects of transgenes. Environ Biosafety Res. 2003;2:9–24. 15615064 10.1051/ebr:2003001

[4] Maillet J, Lopez-Garcia C. What criteria are relevant for predicting the invasive capacity of a new agricultural weed? The case of invasive American species in France. Weed Res. 2000;40:11–26.

[5] Ellstrand NC. Dangerous liaisons? When cultivated plants mate with their wild relatives. Baltimore MD: Johns Hopkins University Press; 2003.

[6] Kilian B, Mammen K, Millet E, Sharma R, Graner A, Salamini F et al Aegilops In: Kole C, editor. Wild Crop Relatives: Genomic and Breeding Resources, Cereals. Springer-Verlag Berlin Heidelberg; 2011 pp.1–76.

[7] van Slageren MW. Wild wheats: a monograph of Aegilops L. and Ambylopyrum (Jaub. & Spach) Eig (Poaceae). Wageningen: Agricultural University; 1994.

[8] Zaharieva M, Monneveux P. Spontaneous hybridization between bread wheat (*Triticum aestivum* L.) and its wild relatives in Europe. Crop Sci. 2006;46:512–527.

[9] Econopouly BF, McKay JK, Westra P, Reid SD, Helm AL, Byrne PF. Phenotypic diversity of *Aegilops cylindrica* (jointed goatgrass) accessions from the western United States under irrigated and dryland conditions. Agric Ecosyst Environ. 2013; 164:244–251.

[10] Rice KJ, Gerlach JD, Dyer A, McKay JK. Evolutionary ecology along invasion fronts of the annual grass *Aegilops triuncialis*. Biol Invasions. 2013;15:2531–2545.

[11] Davy JS, Ditomaso JM, Laca EA. Barb Goatgrass. University of California, Agriculture and Natural Ressources. 2008; publication 1835. Available: http://anrcatalog.ucanr.edu/pdf/8315.pdf.

[12] Westbrooks R. Invasive plants, changing the landscape of America. Federal Interagency Committee for the Management of Noxious and Exotic Weeds (Utah Regional Depository). 1998; paper 490. Available: http://digitalcommons.usu.edu/govdocs/490.

[13] Morrison LA, Riera-Lizarazu O, Crémieux L, Mallory-Smith CA. Jointed goatgrass (*Aegilops cylindrica* Host) x wheat (*Triticum aestivum* L.) hybrids: hybridization dynamics in Oregon wheat fields. Crop Sci. 2002;42:1863–1872.

[14] Schoenenberger N, Felber F, Savova-Bianchi D, Guadagnuolo R. Introgression of wheat DNA markers from A, B and D genomes in early generation progeny of *Aegilops cylindrica* Host x *Triticum aestivum* L. hybrids. Theor Appl Genet. 2005;111:1338–1346. 16133306 10.1007/s00122-005-0063-7

[15] Cifuentes M, Garcia-Agüero V, Benavente E. A Comparative Analysis of Chromosome Pairing at metaphase I in interspecific hybrids between durum wheat (*Triticum turgidum* L) and the most widespread *Aegilops* species. Cytogenet Genome Res. 2010;129:124–132. 10.1159/000313593 20551603

[16] Arrigo N, Guadagnuolo R, Lappe S, Pasche S, Parisod C, Felber F. Gene flow between wheat and wild relatives: empirical evidence from *Aegilops geniculata*, *Ae*. *neglecta* and *Ae*. *triuncialis*. Evol Appl. 2011;4:685–695. 10.1111/j.1752-4571.2011.00191.x 25568015 PMC3352535

[17] Parisod C, Definod C, Sarr A, Arrigo N, Felber F. Genome-specific introgression between wheat and its wild relative *Aegilops triuncialis*. J Evolution Biol. 2013;26: 223–228. 10.1111/jeb.12040 23205963

[18] Pajkovic M, Lappe S, Barman R, Parisod C, Neuenschwander S, Goudet J, et al Wheat alleles introgress into selfing wild relatives: empirical estimates from approximate Bayesian computation in *Aegilops triuncialis*. Mol Ecol. 2014;23:5089–5101. 10.1111/mec.12918 25223217

[19] Weissmann S, Feldman M, Gressel J. Sequence evidence for sporadic intergeneric DNA introgression from wheat into a wild *Aegilops* species. Mol Biol Evol. 2005;22:2055–2062. 15972848 10.1093/molbev/msi196

[20] Parmesan C, Yohe G. A globally coherent fingerprint of climate change impacts across natural systems. Nature. 2003;421:37–42. 12511946 10.1038/nature01286

[21] Hickling R, Roy DB, Hill JK, Fox R, Thomas CD. The distributions of a wide range of taxonomic groups are expanding polewards. Glob Change Biol. 2006;12:450–455.

[22] Devictor V, van Swaay C, Brereton T, Brotons L, Chamberlain D, Heliölä J, et al Differences in the climatic debt of birds and butterflies at a continental scale. Nature Climate Change 2012;2:121–124.

[23] Thomas CD, Cameron A, Green RE, Bakkenes M, Beaumont LJ, Collingham YC, et al Extinction risk from climate change. Nature. 2004; 427:145–147. 14712274 10.1038/nature02121

[24] Pearson RG, Dawson TE. Predicting the impacts of climate change on the distribution of species: are bioclimate envelope models useful? Glob Ecol Biogeogr. 2003;12:361–372.

[25] Cicero C. Barriers to sympatry between avian sibling species (*Paridae*: *Beolophus*) in tenuous secondary contact. Evolution. 2004;58:1573–1587. 15341159 10.1111/j.0014-3820.2004.tb01737.x

[26] Thuiller W, Lavorel S, Araújo MB, Sykes MT, Prentice IC. Climate change threats to plant diversity in Europe. P Natl Acad Sci USA. 2005;102:8245–8250. 10.1073/pnas.0409902102 PMC1140480 15919825

[27] Swenson NG. GIS-based niche models reveal unifying climatic mechanisms that maintain the location of avian hybrid zone locations in a North American suture zone. J Evolution Biol. 2006;19:717–725. 10.1111/j.1420-9101.2005.01066.x 16674568

[28] Kozak KH, Graham CH, Wiens JJ. Integrating GIS-based environmental data into evolutionary biology. Trends Ecol Evol. 2008;23:141–48 10.1016/j.tree.2008.02.001 18291557

[29] Carnaval AC, Hickerson MJ, Haddad CFB, Rodrigues MT, Moritz C. Stability predicts genetic diversity in the Brazilian atlantic forest hotspot. Science. 2009;323:785–789. 10.1126/science.1166955 19197066

[30] Jarvis A, Lane A, Hijmans R. The effect of climate change on crop wild relatives. Agr Ecosyst Environ. 2008;126:13–23.

[31] Nakazato T, Warren DL, Moyle LC. Ecological and geographic modes of species divergence in wild tomatoes. Am J Bot. 2010;97:680–693. 10.3732/ajb.0900216 21622430

[32] Hufford MB, Martı´nez-Meyer E, Gaut BS, Eguiarte LE, Tenaillon MI. Inferences from the historical distribution of wild and domesticated maize provide ecological and evolutionary insight. PLOS One. 2012 11 14 10.1371/journal.pone.0047659 PMC3498274 23155371

[33] Elith J, Graham CH, Anderson RP, Dudı´k M, Ferrier S, Guisan A, et al Novel methods improve prediction of species’ distributions from occurrence data. Ecography. 2006;29: 129–151.

[34] Elith J, Graham C. Do they? How do they? WHY do they differ? On finding reasons for differing performances of species distribution models. Ecography. 2009;32: 66–77.

[35] Phillips SJ, Dudı´k M. Modeling of species distributions with MaxEnt: new extensions and a comprehensive evaluation. Ecography. 2008;31:161–175.

[36] Elith J, Phillips SJ, Hastie T, Dudı´k M, Chee YE, Yates CJ. A statistical explanation of MaxEnt for ecologists. Divers Distrib. 2011;17:43–57.

[37] Peterson AT. Ecological niche conservatism: a time-structured review of evidence. J Biogeogr. 2011;38:817–827.

[38] Maxted N, Kell SP. Establishment of a Global Network for the In Situ Conservation of Crop Wild Relatives: Status and Needs. Background study—FAO. 2009; paper N°39. Available: http://www.fao.org/docrep/013/i1500e/i1500e18a.pdf.

[39] Phillips SJ, Dudík M, Elith J, Graham C, Lehmann A, Leathwick J, et al Sample selection bias and presence-only models of species distributions: implications for background and pseudo-absence data. Ecol Appl. 2009;19:181–97. 19323182 10.1890/07-2153.1

[40] Syfert MM, Smith MJ, Coomes DA. The effects of sampling bias and model complexity on the predictive performance of MaxEnt species distribution models. PLOS One. 2013 2 14 10.1371/journal.pone.0055158 PMC3573023 23457462

[41] Hufford MB, Martínez-Meyer E, Gaut BS, Eguiarte LE, Tenaillon MI. Inferences from the historical distribution of wild and domesticated maize provide ecological and evolutionary insight. PLOS One 2012;7:e47659 10.1371/journal.pone.0047659 23155371 PMC3498274

[42] Phillips SJ. A brief tutorial on MaxEnt. AT&T Labs-Research, Princeton University, and the Center for Biodiversity and Conservation, American Museum of Natural History 2011.

[43] Hijmans RJ, Cameron SE, Parra JL, Jones PG, Jarvis A. Very high resolution interpolated climate surfaces for global land areas. Int J Climatol. 2005;25:1965–1978.

[44] Qin D, Plattner GK, Tignor, Allen SK, Boschung J, Nauels A, et al Climate change 2013: The physical science basis. Stocker T., editor. Cambridge, UK, and New York: Cambridge University Press; 2014.

[45] van Vuuren DP, Edmonds J, Kainuma MLT, Riahi K, Thomson A, Matsui T, et al Representative concentration pathways: An overview. Climatic Change. 2011;109:5–31.

[46] Warren D, Seifert SS. Environmental niche modeling in MaxEnt: the importance of model complexity and the performance of model selection criteria. Ecol Appl. 2011;21:335–342. 21563566 10.1890/10-1171.1

[47] Elith J, Kearney M, Phillips SJ. The art of modelling range-shifting species. Methods Ecol Evol. 2010;1:330–342.

[48] Raes N, Ter Steege H. A null-model for significance testing of presence only species distribution models. Ecography. 2007;30: 727–736.

[49] Liu C, Berry PM, Dawson TP, Pearson RG. Selecting thresholds of occurrence in the prediction of species distributions. Ecography 2005;28:385–393.

[50] Merow C, Smith MJ, Silander JA Jr. A practical guide to MaxEnt for modeling species’ distributions: what it does, and why inputs and settings matter. Ecography. 2013;36:1058–1069.

[51] Datta SC, Evenari M, Gutterman Y. The infuence of the origin of the mother plant on yield and germination of their caryopses in *Aegilops ovata* L. Planta. 1972;105:155–164. 10.1007/BF00385574 24477754

[52] Dyer AR, Brown CS, Espeland EK, McKay JK, Meimberg H, Rice KJ. The role of adaptive trans-generational plasticity in biological invasions of plants. Evol Appl. 2010;3:179–192. 10.1111/j.1752-4571.2010.00118.x 25567918 PMC3352481

[53] Fandrich L, Mallory-Smith C. Jointed goatgrass (Aegilops cylindrica) seed germination and production varies by spikelet position on the spike. Weed Sci. 2006;54:443–451.

[54] Marañon T. Variations in seed size and germination in three *Aegilops* species. Seed Sci Technol. 1989;17: 583–588.

[55] Jump AS, Mátyás C, Peñuelas J. The altitude-for-latitude disparity in the range retractions of woody species. Trends Ecol Evol. 2009;24:694–701. 10.1016/j.tree.2009.06.007 19695735

[56] Zaharieva M, Dimov A, Stankova P, David J, Monneveux P. Morphological diversity and potential interest for wheat improvement of three Aegilops L. species from Bulgaria. Genet Resour Crop Ev. 2003;50:507–517.

[57] Jia J, Zhao S, Kong X, Li Y, Zhao G, He W, et al *Aegilops tauschii* draft genome sequence reveals a gene repertoire for wheat adaptation. Nature. 2013;496:91–95. 10.1038/nature12028 23535592

[58] Badaeva ED, Amosova AV, Muravenko OV, Samatadze TE, Chikida NN, Zelenin AV, et al Genome differentiation in *Aegilops*. 3. Evolution of the D-genome cluster. Plant Syst Evol. 2002;231:163–190.

[59] Donald WW. Vernalization requirements for flowering of Jointed Goatgrass (Aegilops cylindrica). Weed Sci. 1984;32: 631–637.

[60] Perrino EV, Wagensommera RP, Medaglic P. *Aegilops* (Poaceae) in Italy: taxonomy, geographical distribution, ecology, vulnerability and conservation. Syst Biodivers. 2014;12: 1–19.

[61] Torres NM, De Marco Junior P, Santos T, Silveira L, de Almeida Jacomo AT, Diniz‐Filho JA. Can species distribution modelling provide estimates of population densities? A case study with jaguars in the Neotropics. Divers Distrib. 2012;18: 615–627.

[62] Zohary D, Harlan JR, Vardi A. The wild diploid progenitors of wheat and their breeding value. Euphytica. 1969;18:58–65.

[63] Lyons KG, Shapiro AM, Schwartz MW. Distribution and ecotypic variation of the invasive annual barb goatgrass (*Aegilops triuncialis*) on serpentine soil. Invasive Plant Sci Manage. 2010;3: 376–389.

[64] Beauregard F. de Blois S. Beyond a climate-centric view of plant distribution: Edaphic variables add value to distribution models. PLOS One. 2014 3 21 10.1371/journal.pone.0092642 PMC3962442 24658097

[65] Lyon DJ, Baltensperger DD, Rush IG. Viability, germination, and emergence of cattle-fed jointed goatgrass seed. J Prod Agric. 1992;5:282–285.

[66] Peters A, Johnson DE, George MR. Barb goatgrass: a threat to California rangelands. Rangelands. 1996;18: 8–10.

[67] Thomson DM. Do source—sink dynamics promote the spread of an invasive grass into a novel habitat? Ecology. 2007;88:3126–3134. 18229846 10.1890/06-1463.1

[68] Donald WW, Ogg AG. Biology and control of jointed goatgrass (*Aegilops cylindrica*), a review. Weed Technol. 1991;5:3–17.

[69] DiTomaso JM, Healy EA. Weeds of California and Other Western States. Oakland: University of California, Agriculture and Natural Resources; 2007.

[70] Cain ML, Milligan BG, Strand A. Long-distance dispersal in plant populations. Am J Bot. 2000;87:1217–1227. 10991892

[71] Zaharieva M, Gaulin E, Havaux M, Acevedo E, Monneveux P. Drought and heat responses in the wild wheat relative *Aegilops geniculata* Roth: potential interest for wheat improvement. Crop Sci. 2001;41: 1321–1329.

[72] Arrigo N, Felber F, Parisod C, Buerki S, Alvarez N, David J, Guadagnuolo R. Origin and expansion of the allotetraploid *Aegilops geniculata*, a wild relative of wheat. New Phytol. 2010;187:1170–1180. 10.1111/j.1469-8137.2010.03328.x 20561204

[73] Martínez‐Meyer E, Peterson AT. Conservatism of ecological niche characteristics in North American plant species over the Pleistocene-to-Recent transition. J Biogeogr. 2006;33:1779–1789.

[74] Davis MB. Shaw RG. Range shifts and adaptive responses to Quaternary climate change. Science. 2001;292:673–679. 11326089 10.1126/science.292.5517.673

[75] Zohary D. The colonizer species in the wheat group In: Baker HG, Stebbins GL, editors. The genetics of colonizing species. New York/ London: Academic Press; 1965 pp. 403–419.

[76] Zaharieva M, Prosperi JM, Monneveux P. Ecological distribution and species diversity of Aegilops L. genus in Bulgaria. Biodivers Conserv. 2004;13: 2319–37.

[77] Hammer K. Vorarbeiten zur Monographischen Darstellung von Wildpflanzensortimenten: *Aegilops* L. Kulturpflanze. 1980;28:33–180. German.

[78] Zohary D, Feldman M. Hybridization between amphidiploids and the evolution of polyploids in the wheat (*Aegilops-Triticum*). Evolution. 1962;16:44–61.

[79] Meimberg H, Rice KJ, Milan NF, Collins C, Njoku CC, McKay JK. Multiple origins promote the ecological amplitude of allopolyploid Aegilops (Poaceae). Am J Bot. 2009;96:1262–1273. 10.3732/ajb.0800345 21628275

[80] te Beest M, Le Roux JJ, Richardson DM, Brysting AK, Suda J, Kubešová M, et al The more the better? The role of polyploidy in facilitating plant invasions. Ann Bot. 2011;109:19–45. 10.1093/aob/mcr277 22040744 PMC3241594

[81] Mahjoub A, Rouaissi M, Mguis K, El Gharbi MS, El Gazzah M, Ben Brahim N. Agromorphological Variation in Spontaneous *Aegilops geniculata* Roth. Populations Suitable for Mediterranean Conditions. World J Agric Sci. 2008;4:737–744.

[82] Wang AB, Tan DY, Baskin CC, Baskin JM. Effect of seed position in spikelet on life history of *Eremopyrum distans* (Poaceae) from the cold desert of northwest China. Ann Bot. 2010;106:95–105. 10.1093/aob/mcq089 20460387 PMC2889797

[83] Meimberg H, Milan NF, Karatassiou M, Espeland EK, McKay JK, Rice K. Patterns of introduction and adaptation during the invasion of *Aegilops triuncialis* (Poaceae) into Californian serpentine soils. Mol Ecol. 2010;19:5308–5319. 10.1111/j.1365-294X.2010.04875.x 20977511

[84] Gandhi HT, Vales MI, Mallory-Smith CA, Riera-Lizarazu O. Genetic structure of *Aegilops cylindrica* in its native range and in the United States of America. Theor Appl Genet. 2009;119:1013–1025. 10.1007/s00122-009-1105-3 19618161

[85] Dlugosch KM, Parker IM. Founding events in species invasions: genetic variation, adaptive evolution, and the role of multiple introductions. Mol. Ecol. 2008;17:431–449. 17908213 10.1111/j.1365-294X.2007.03538.x

[86] Schneider A, Molnár I, Molnár-Láng M. Utilisation of *Aegilops* (goatgrass) species to widen the genetic diversity of cultivated wheat. Euphytica 2008;163:1–19.

[87] Maxted N, White K, Valkoun J, Konopka J, Hargreaves S. Towards a conservation strategy for *Aegilops* species. Plant Genet Resour. 2008;6:126–141.

[88] International Union for Conservation of Nature and Natural Resources. IUCN Red List Categories and Criteria: Version 3.1. IUCN, Gland, Switzerland and Cambridge, UK; 2001 Available: http://www.iucnredlist.org/technical-documents/categories-and-criteria/2001-categories-criteria.

[89] Guadagnuolo R, Savova-Bianchi D, Felber F. Gene flow from wheat (*Triticum aestivum* L.) to jointed goatgrass (*Aegilops cylindrica* Host.), as revealed by RAPD and microsatellite markers. Theor Appl Genet. 2001;103: 1–8.

[90] David JL, Benavente E, Brès-Patry C, Dusautoir J, Echaide M. Biological relevance of polyploidy: ecology to genomics. Are neopolyploids a likely route for a transgene walk to the wild? The *Aegilops ovata* x *Triticum turgidum durum* case. Biol J of the Linn Soc. 2004;82:503–510.

[91] Loureiro I, Escorial C, García-Baudín JM, Chueca MC. Hybridisation between wheat and *Aegilops geniculata* and hybrid fertility for potential herbicide resistance transfer. Weed Res. 2008;48: 561–570.

[92] Loureiro I, Escorial C, García-Baudín J M, Chueca M C.Spontaneous wheat-*Aegilops biuncialis*, *Ae*. *geniculata* and *Ae*. *triuncialis* amphiploid production, a potential way of gene transference. Span J Agric Res. 2009;7: 614–620.

[93] Waines JG, Hegde SG. Intraspecific gene flow in bread wheat as affected by reproductive biology and pollination ecology of wheat flowers.Crop Sci. 2003;43:451–463.

[94] Snyder JR, Mallory-Smith CA, Balter S, Hansen JL, Zemetra RS. Seed production on *Triticum aestivum* by *Aegilops cylindrica* hybrids in the field. Weed Sci. 2000;48:588–593.

[95] Saini HS, Sedgley M, Aspinall D. Development anatomy in wheat of male sterility induced by heat stress, water defi cit or abscisic acid. Aust. J. Plant Physiol. 1984;11:243–253.

[96] Sakata T, Takahashi H, Nishiyama I, Higashitani A. Effects of high temperature on the development of pollen mother cells and microspores in barley *Hordeum vulgare* L. J Plant Res. 2000;113:395–402.

[97] Endo M, Tsuchiya T, Hamada K, Kawamura S, Yano K, Ohshima M, et al High temperatures cause male sterility in rice plants with transcriptional alterations during pollen development. Plant Cell Physiol. 2009; 50:1911–1922. 10.1093/pcp/pcp135 19808807

[98] Farooq M, Bramley H, Palta JA, Siddique KH. Heat stress in wheat during reproductive and grain-filling phases. Crit Rev Plant Sci. 2011; 30:491–507.

[99] Sakata T, Higashitani A. Male sterility accompanied with abnormal anther development in plants—genes and environmental stresses with special reference to high temperature injury. Int J Plant Dev Biol, 2008;2: 42–51.

[100] Lukac M, Gooding MJ, Griffiths S, Jones HE. Asynchronous flowering and within-plant flowering diversity in wheat and the implications for crop resilience to heat. Ann Bot-London. 2012; 109:843–850. 10.1093/aob/mcr308 PMC3286278 22186277

[101] Hufbauer RA, Rutschmann A, Serrate B, Vermeil de Conchard H, Facon B. Role of propagule pressure in colonization success: disentangling the relative importance of demographic, genetic and habitat effects. J Evol Biol. 2013;26: 1691–1699. 10.1111/jeb.12167 23724778

[102] Syngenta. 2013 Aug 29. Available: http://www.syngentacropprotection.com/news_releases/news.aspx?id=177068

[103] David JL, Pham JL. Rapid changes in pollen production in experimental outcrossing populations of wheat. J Evol Biol. 1993;6: 659–676.

[104] Rounsevell MDA, Ewert F, Reginster I, Leemans R, Carter TR. Future scenarios of European agricultural land use.II. Projecting changes in cropland and grassland. Agric Ecosyst Environ. 2005;107:117–135.

[105] Hurtt GC, Chini LP, Frolking S, Betts RA, Feddema J, Fischer G, et al Harmonization of land-use scenarios for the period 1500–2100: 600 years of global gridded annual land-use transitions, wood harvest, and resulting secondary lands. Climatic Change. 2011;109:117–161.

[106] Tilman D, Balzer C, Hill J, Befort BL. Global food demand and the sustainable intensification of agriculture. P Natl Acad Sci USA. 2011;108:20260–20264. 10.1073/pnas.1116437108 PMC3250154 22106295

[107] Wise M, Calvin K, Thomson A, Clarke L, Bond-Lamberty B, Sands R, et al Implications of limiting CO2 concentrations for land use and energy. Science. 2009;324:1183–1186. 10.1126/science.1168475 19478180

[108] Harfoot M, Tittensor DP, Newbold T, McInerny G, Smith MJ, Scharlemann JP. Integrated assessment models for ecologists: the present and the future. Global Ecol Biogeogr. 2014;23:124–143.

[109] Audsley E, Trnka M, Sabaté S, Maspons J, Sanchez A, Sandars D, et al Interactively modelling land profitability to estimate European agricultural and forest land use under future scenarios of climate, socio-economics and adaptation. Climatic Change. 2014;128:215–227.

[110] Guisan A, Thuiller W. Predicting species distribution: offering more than simple habitat models. Ecol Lett. 2005;8:993–1009. 10.1111/j.1461-0248.2005.00792.x 34517687

[111] Alexandratos N, Bruinsma J. World Agriculture Towards 2030/2050: The 2012 Revision. ESA Work Pap. 2012; paper N°12–03. Available: http://www.fao.org/docrep/016/ap106e/ap106e.pdf.

[112] Meeséan A, Sweet J. SIGMEA (Sustainable Introduction of GM crops into European Agriculture. 2012. Available: http://cordis.europa.eu/docs/publications/1267/126792601-6_en.pdf.

