# Supplementary material for: Potential Implications of Climate Change on Aegilops Species Distribution: Sympatry of These Crop Wild Relatives with the Major European Crop Triticum aestivum and Conservation Issues
Source: PLoS One. 2016 Apr 21;11(4):e0153974. doi: 10.1371/journal.pone.0153974 (PMC4839726; doi:10.1371/journal.pone.0153974)

**S1 Appendix**

**Table A1S1**. Net number of cleaned geo-referenced occurrences per species including duplicates. Sources: Gbif stands for Global Biodiversity Information Facility, CBNs for 'Conservatoires Botaniques Nationaux' (France) and MWVS for M. W. van Slageren.

| Source | *Ae. biuncialis* | *Ae. cylindrica* | *Ae.geniculata* | *Ae.neglecta* | *Ae.triuncialis* | *Ae.ventricosa* |
|---|---|---|---|---|---|---|
| Gbif | 637 | 537 | 2009 | 774 | 1760 | 146 |
| CBNs | 56 | 145 | 530 | 464 | 539 | 29 |
| MWVS | 104 | 107 | 136 | 50 | 157 | 9 |
| Total | 797 | 789 | 2675 | 1288 | 2456 | 184 |

**Table A1S2.** Training sample and background sizes. Background size corresponds to the number of 0.5° x 0.5° pixels included in the training grids (shown below). The fraction of background cells corresponding to occurrence points (after duplicate removal) is given in parenthesis. The extent of the background and the projection grids is also given ($x_{min}$, $x_{max}$; $y_{min}$, $y_{max}$; in decimal degrees).

| Species | N | Background size | Extent $_{Background}$ | Extent $_{Projection}$ |
|---|---|---|---|---|
| *Ae. biuncialis* Vis. | 255 | 2136 (12%) | ( -7, 53 ; 30, 49) | (-10, 71; 30, 55) |
| *Ae. cylindrica* Host | 289 | 2674 (11%) | (-10, 71; 30, 49) | " |
| *Ae. neglecta* Req. *ex* Bertol. | 284 | 2049 (14%) | (-10, 53; 30, 47) | " |
| *Ae. geniculata* Roth. | 495 | 2130 (23%) | (-10, 53; 30, 47) | " |
| *Ae. triuncialis* L. | 621 | 2781 (22%) | (-10, 71; 30, 49) | " |
| *Ae. ventricosa* Tausch | 101 | 1679 (6%) | (-10, 37; 30, 49) | " |

**Figure A1S1.** Training grids**.** For each species, the top and bottom panel includes occurrences before and after duplicate removal, respectively.

## *Ae. biuncialis*

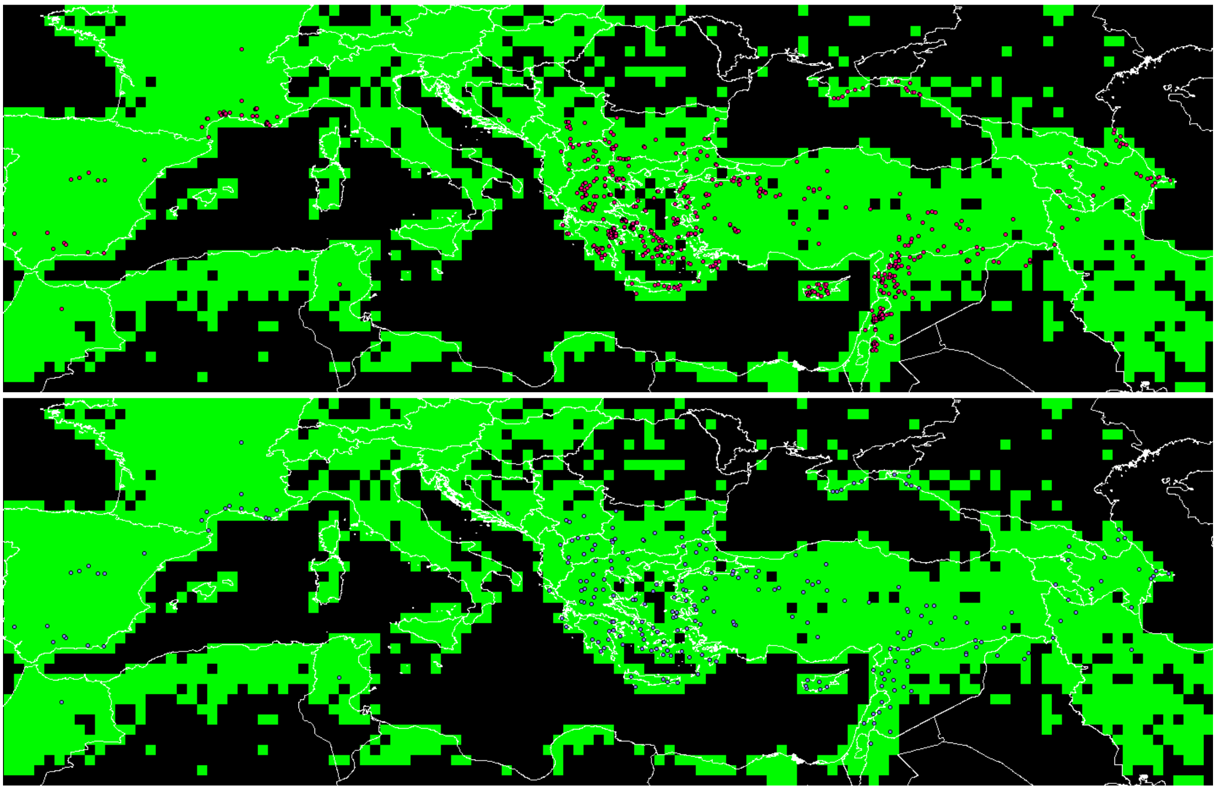

*Ae. cylindrica*

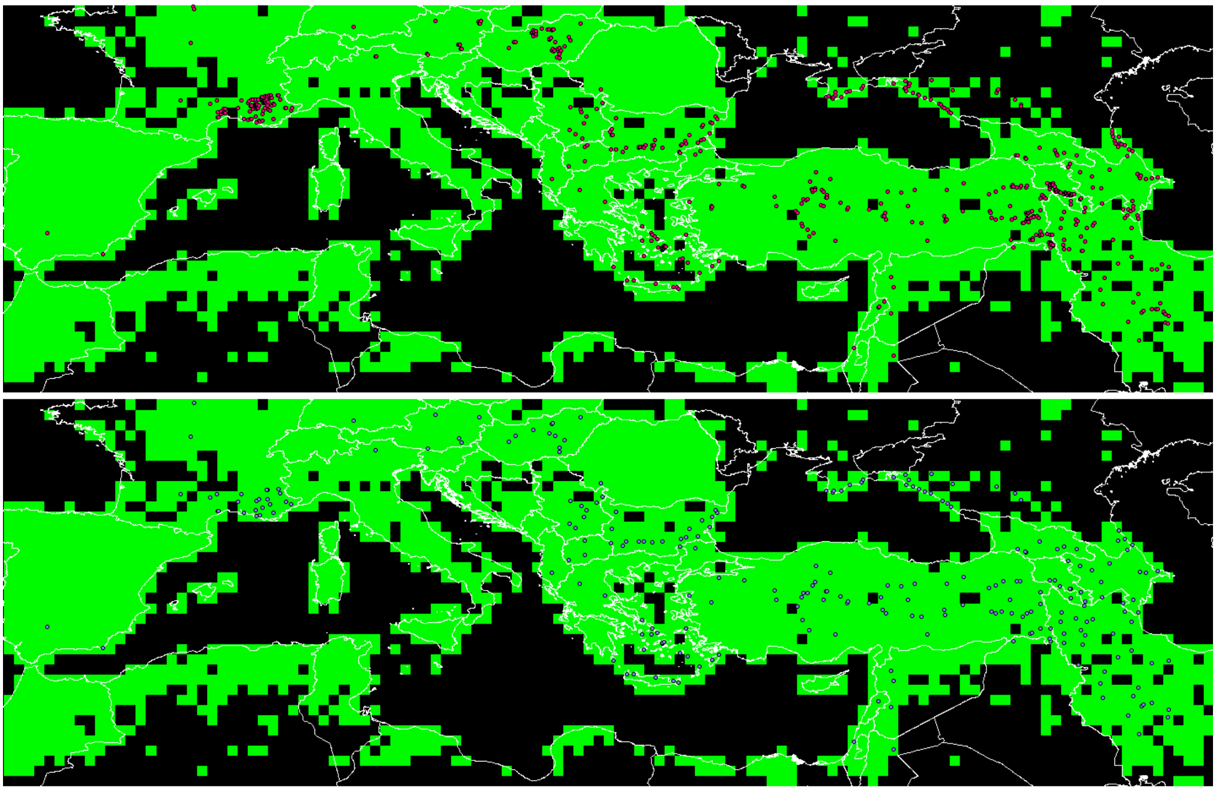

*Ae. geniculata*

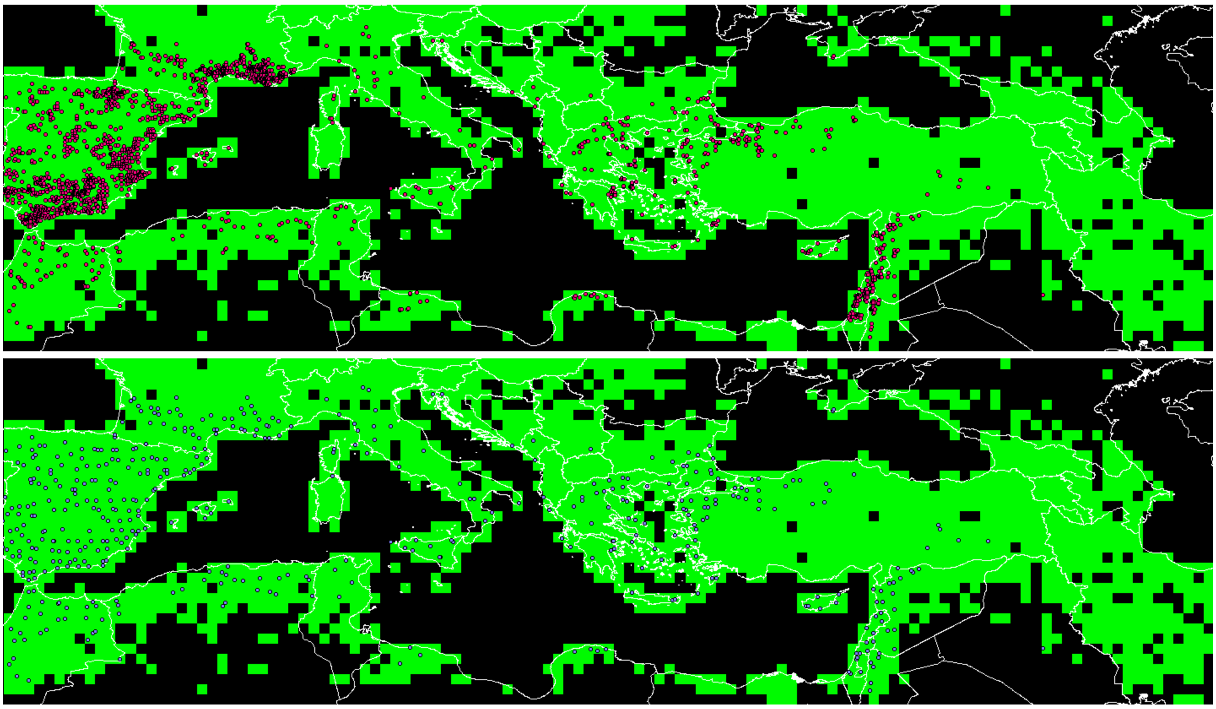

*Ae. neglecta*

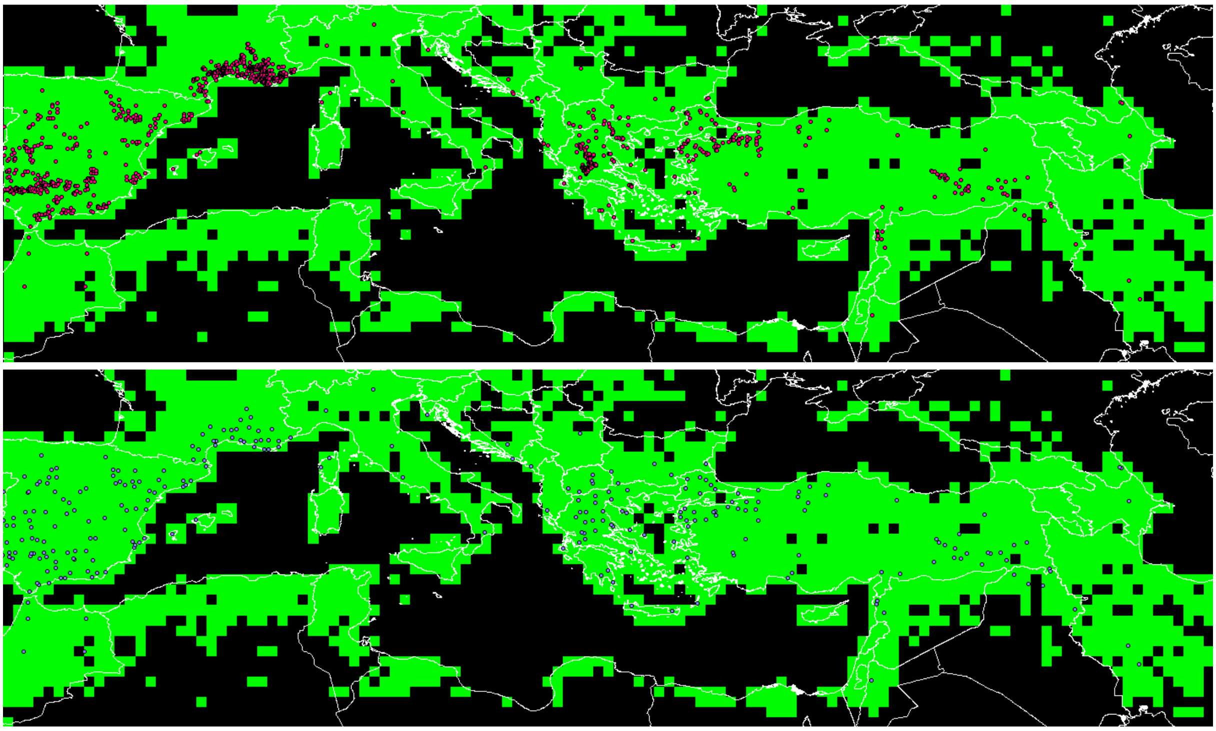

*Ae. triuncialis*

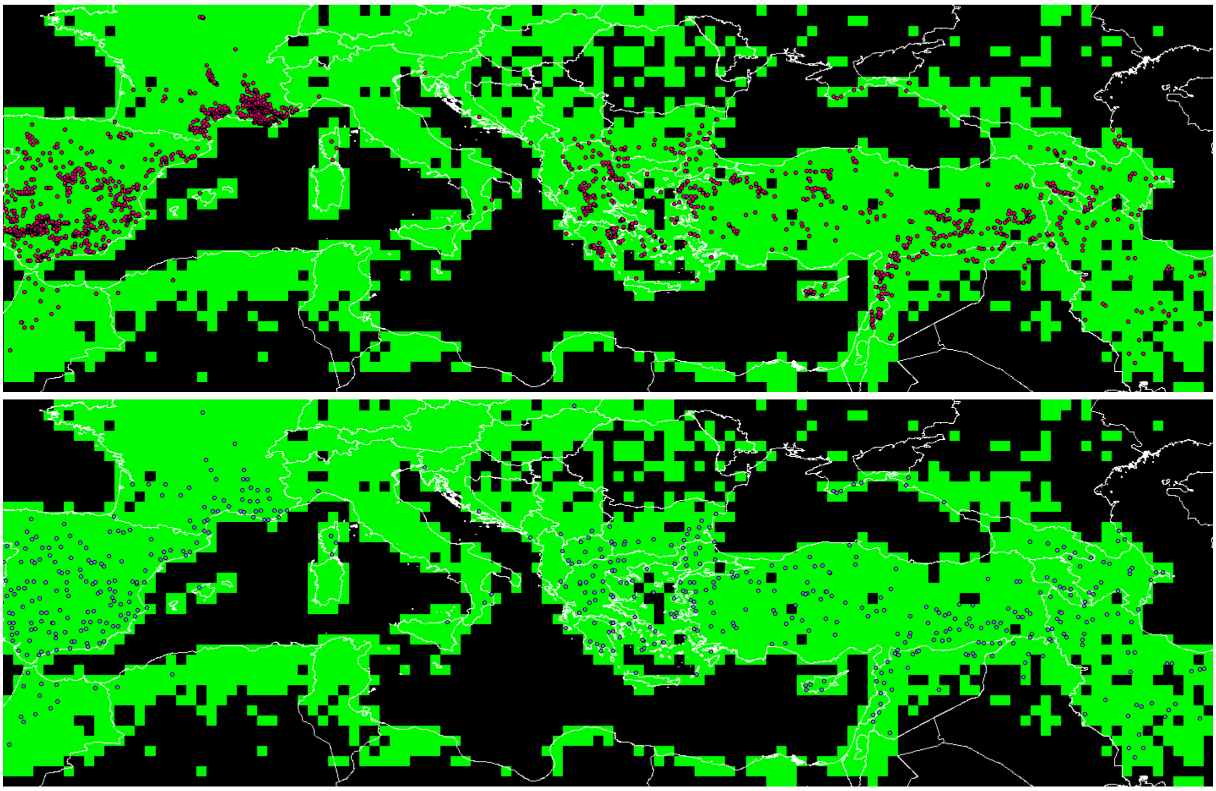

### *Ae. ventricosa*

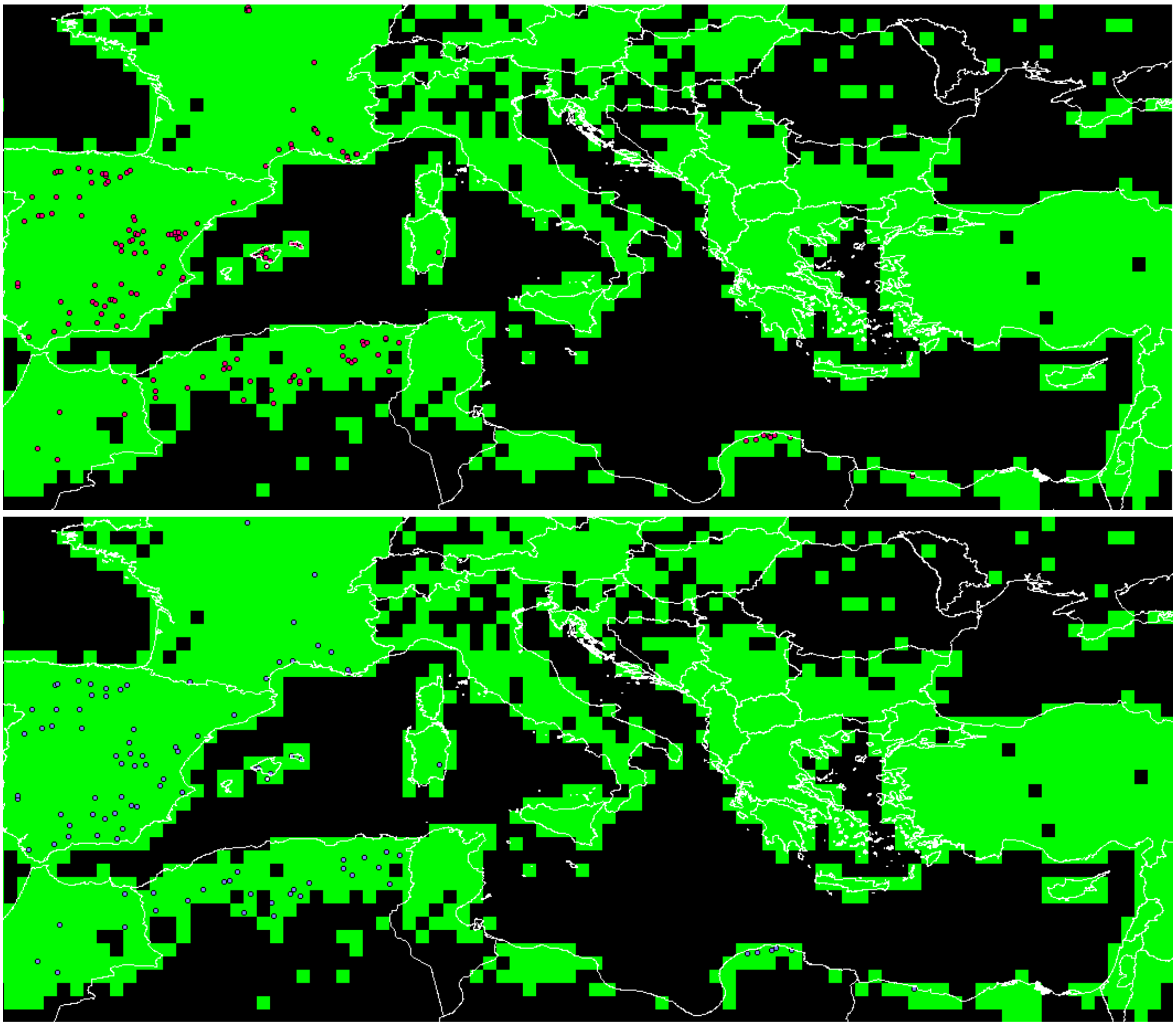

Supplement: S1 Appendix — (PDF) [file pone.0153974.s001.pdf]
