# Supplementary material for: Potential Implications of Climate Change on Aegilops Species Distribution: Sympatry of These Crop Wild Relatives with the Major European Crop Triticum aestivum and Conservation Issues
Source: PLoS One. 2016 Apr 21;11(4):e0153974. doi: 10.1371/journal.pone.0153974 (PMC4839726; doi:10.1371/journal.pone.0153974)
Supplement: S2 Appendix — (PDF) [file pone.0153974.s002.pdf]

S2 Appendix: sample means and variable contribution

The contribution of the bioclimatic variables was examined using mean values of three summary statistics provided by MaxEnt when performing ten-fold cross-validations (Phillips, 2011): 1) Percent contribution (hereafter PC): the net gain in fit associated with each variable is summed over all algorithm steps during the training process and converted into percentages at the end; 2) Permutation importance (hereafter PI): successively for each variable in the final model, environmental values in the background (including occurrence locations) are randomly permuted, and the loss in fit indicates how heavily the model depends on that variable; 3) Individual Variable Contribution (hereafter IC): each variable is used individually to build a species model and the fit of the model to the data is gauged for each variable separately, relative to the complete/final model.

We retained as leading candidate variables those for which all three statistics ranked in the top five highest values; the variable for which two of the three statistics were ranked in the top five were considered as important contributors (Hufford et al. 2012). As the permutation importance (PI) reflects how heavily a model depends on a particular variable, we also paid attention to variables ranking in the top three highest values, regardless of their rank for the two other statistics.

Most recurrent contributing variables

All six species belonging to the same genus, and as the background largely overlapped (S1 Appendix), recurrence in variable contribution was expected. The temperature variables related to the coldest periods either through BIO6 (coldest month) and/or BIO11 (coldest quarter)—two highly correlated variables (**Table A2S3**)—contributed substantially to most of the species models (**Table A2S2**). For these variables, *Ae. cylindrica* was the only species presenting lower sample mean values than averaged across its background (**Figure A2S1**). It was also characterized with lower sample means than all other species (**Table A2S1**). In turn, the precipitation variables related to the warmest/driest periods either through BIO18 (warmest quarter) and/or BIO17 (driest quarter), but

also through BIO14 (driest month)—three highly correlated variables (**Table A2S3**)—appeared to be the most recurrent contributing variables across the species models (**Table A2S2**).

Cited references

Phillips, S. 2011. A brief tutorial on MaxEnt. AT&T Research. Available at: https://www.cs.princeton.edu/~schapire/maxent/tutorial/.

Hufford MB, Martínez-Meyer E, Gaut BS, Eguiarte LE, Tenaillon MI. Inferences from the historical distribution of wild and domesticated maize provide ecological and evolutionary insight. PLoS One 2012;7:e47659.

Table A2S1. Training sample means and standard deviations of training points (SD) for the BIOclim variables (BIO). The value in parenthesis corresponds to 1.96 times the standard error of the mean.

BIO[a]	*Ae. geniculata* Mean	SD	*Ae. neglecta* Mean	SD	*Ae. ventricosa* Mean	SD	*Ae. biuncialis* Mean	SD	*Ae. triuncialis* Mean	SD	*Ae. cylindrica* Mean	SD
**1.** Annual Mean Temperature	14.4 (0.2)	2.6	13.7 (0.3)	2.3	13.7 (0.5)	2.4	14.1 (0.4)	3.0	13.1 (0.2)	3.1	11.0 (0.4)	3.5
**2.** Mean Diurnal Range	10.3 (0.2)	1.8	10.3 (0.1)	1.2	10.7 (0.3)	1.6	10.0 (0.3)	2.2	10.9 (0.1)	1.8	10.5 (0.3)	2.2
**3.** Isothermality	37.5 (0.3)	3.6	35.9 (0.3)	2.8	38.3 (0.6)	2.9	34.1 (0.5)	4.0	35.3 (0.3)	3.8	32.4 (0.4)	3.3
**4.** Temperature Seasonality	629.8 (6.6)	75.4	675.9 (13.3)	114.3	625.2 (13.9)	71.3	720.0 (14.8)	120.8	736.0 (11.1)	141.5	806.2 (15.1)	130.9
**5.** Max Temperature of Warmest Month	29.7 (0.3)	3.0	29.7 (0.4)	3.4	29.5 (0.6)	3.2	30.1 (0.4)	3.4	30.1 (0.3)	3.6	28.5 (0.4)	3.6
**6.** Min Temperature of Coldest Month	2.2 (0.3)	3.0	0.9 (0.3)	2.8	1.5 (0.5)	2.6	0.7 (0.5)	4.3	-0.9 (0.4)	4.6	-4.0 (0.6)	5.2
**7.** Temperature Annual Range	27.5 (0.3)	3.5	28.8 (0.4)	3.8	28.0 (0.7)	3.6	29.4 (0.6)	5.0	31.0 (0.4)	5.1	32.5 (0.6)	5.6
**8.** Mean Temperature of Wettest Quarter	10.3 (0.3)	3.1	9.5 (0.4)	3.5	10.6 (0.6)	3.2	8.9 (0.4)	3.6	9.1 (0.3)	3.8	9.4 (0.6)	5.1
**9.** Mean Temperature of Driest Quarter	21.2 (0.4)	4.2	20.8 (0.6)	4.8	20.9 (0.7)	3.7	21.4 (0.6)	4.7	20.4 (0.5)	5.9	15.9 (1.0)	8.7
**10.** Mean Temperature of Warmest Quarter	22.3 (0.2)	2.5	22.1 (0.3)	2.7	21.7 (0.5)	2.6	22.9 (0.3)	2.8	22.2 (0.2)	3.1	20.8 (0.4)	3.3
**11.** Mean Temperature of Coldest Quarter	6.9 (0.3)	2.9	5.6 (0.3)	2.8	6.4 (0.5)	2.6	5.4 (0.5)	3.9	4.2 (0.3)	4.2	1.0 (0.5)	4.4
**12.** Annual Precipitation	605.6 (14.6)	165.8	633.8 (15.6)	133.8	521.0 (32.5)	166.7	582.1 (20.6)	168.2	562.8 (12.0)	152.8	569.5 (20.9)	180.9
**13.** Precipitation of Wettest month	88.4 (2.8)	31.9	90.7 (3.1)	26.9	69.5 (4.5)	23.0	95.1 (4.8)	39.0	84.2 (2.2)	28.6	83.2 (3.3)	28.9
**14.** Precipitation of Driest Month	16 (1.3)	14.6	16.4 (1.3)	11.3	14.4 (2.6)	13.3	13.6 (1.7)	14.1	13.9 (1.0)	12.3	18.9 (1.9)	16.6
**15.** Precipitation Seasonality	50.8 (2.3)	25.6	47.5 (2.1)	18.4	43.5 (3.7)	19.2	58.8 (3.4)	27.7	54.0 (1.8)	23.4	49.5 (2.7)	23.4
**16.** Precipitation of Wettest Quarter	237.7 (7.4)	83.7	245.1 (8.6)	73.8	187.7 (12.2)	62.6	251.8 (12.3)	100.3	226.2 (6.1)	77.9	221.3 (9.0)	78.3
**17.** Precipitation of Driest Quarter	73.7 (4.6)	51.7	75.5 (4.8)	41.0	63.5 (8.7)	44.7	60.0 (5.6)	45.6	65.5 (3.4)	43.2	92.0 (6.4)	55.2
**18.** Precipitation of Warmest Quarter	66.0 (4.7)	53.1	67.6 (5.1)	44.1	72.2 (9.2)	47.3	51.0 (6.3)	51.0	55.1 (3.8)	47.9	67.5 (8.3)	71.7
**19.** Precipitation of Coldest Quarter	210.5(7.7)	86.9	215.3 (9.4)	81.1	160.3 (12)	61.6	224.3 (12.2)	99.7	192.6 (6.9)	87.9	168.2 (8.6)	75.0

a : BIOclim variables are derived from monthly temperature and precipitation values. A full description is available at: http://www.worldclim.org/bioclim and http://www.rforge.net/doc/packages/climates/bioclim.html. Temperature variables (BIO1 to BIO11) are in °C, except for BIO3; BIO3 = BIO2/BIO7 *100. BIO2 = mean of monthly (maximum temperature – minimum temperature), BIO4 = standard deviation of monthly temperature*100. Precipitation variables (BIO12 to BIO19) are in mm, except for BIO15; BIO15 = coefficient of variation of monthly precipitation. Sample sizes are available in the S1 Appendix (A1S1 Table).

Table A2S2. Variable contribution. Column headings: PC, PI and IC refer to the percent contribution, permutation importance and individual variable contribution, respectively. These summary statistics were averaged across the 10-fold cross validation runs and are all expressed in percentages. Grey boxes include variables for which all three statistics ranked consistently in the top five highest values; in italics are variables for which two of the three statistics were in the top five. Underlined PI values were in the top three. See Table A2S1 for variable definitions.

	Ae. geniculata			***Ae. neglecta***			***Ae. ventricosa***			***Ae. biuncialis***			***Ae. triuncialis***			***Ae. cylindrica***		
**BIO**	PC	PI	IC	PC	PI	IC	PC	PI	IC	PC	PI	IC	PC	PI	IC	PC	PI	IC
**1**	0.6	3.0	29.0	0.8	0.2	24.0	7.7	11.6	11.9	2.7	6.7	18.6	2.6	1.0	19.6	1.6	0.1	22.0
**2**	2.1	2.0	5.9	14.3	2.6	22.6	1.2	1.4	7.2	3.4	0.3	4.5	11.0	7.1	18.2	1.3	0.7	8.6
**3**	3.0	1.3	16.7	3.9	1.5	13.6	9.5	0.7	20.0	14.2	3.0	8.1	1.1	0.5	9.2	***28.1***	2.3	***39.1***
**4**	***27.3***	1.8	***45.0***	2.4	2.5	9.3	14.8	5.0	10.0	2.0	5.6	2.0	1.5	0.7	9.0	**15.1**	**10.2**	**24.0**
**5**	0.9	0.8	16.4	0.2	0.1	10.4	0.3	0.0	6.4	***6.7***	2.0	***21.2***	3.6	0.0	19.4	1.2	0.1	11.3
**6**	**26.6**	**20.8**	**47.7**	**15.5**	**9.4**	**25.1**	**14.6**	**12.1**	**25.4**	6.2	**10.4**	8.4	3.6	8.3	9.1	6.2	8.3	34.0
**7**	0.8	0.6	27.1	1.1	3.4	10.0	1.2	1.2	0.5	3.7	3.7	2.1	0.6	0.0	11.9	0.4	0.3	13.0
**8**	1.8	2.7	16.7	2.2	3.0	5.5	1.3	0.4	7.0	5.9	1.0	12.4	8.4	2.4	19.0	6.4	2.5	5.5
**9**	***4.7***	1.2	***31.1***	3.9	1.0	14.0	1.1	1.6	19.1	1.6	4.6	19.9	1.1	0.6	19.9	5.3	8.8	12.5
**10**	0.3	0.0	18.8	0.4	0.0	11.6	1.8	0.0	6.0	1.5	0.0	18.7	0.2	0.0	12.7	0.1	0.1	9.8
**11**	**10.1**	**4.7**	**47.1**	1.4	0.3	25.8	5.5	0.7	22.5	2.2	5.4	9.4	2.1	6.3	15.5	**14.5**	**19.4**	**32.1**
**12**	10.2	2.6	16.4	**28.0**	**20.1**	**33.8**	***11.3***	3.5	***25.1***	8.8	4.0	16.9	9.6	2.0	40.5	4.6	0.9	19.0
**13**	0.8	0.7	9.5	0.4	0.1	14.0	10.1	0.5	25.9	2.7	**10.7**	6.1	1.0	1.0	26.7	0.3	2.4	13.2
**14**	1.8	**23.2**	7.7	2.4	6.1	22.0	9.2	**31.6**	19.8	2.6	10.2	16.5	1.1	***15.1***	***22.3***	1.1	**16.0**	6.3
**15**	1.5	2.1	0.7	3.7	2.3	10.0	2.7	5.0	4.7	7.6	3.6	13.0	3.9	3.5	11.0	2.2	9.5	5.1
**16**	3.2	2.3	9.9	0.3	0.1	14.0	3.3	1.3	26.4	1.2	2.6	5.4	**20.7**	**8.6**	**28.2**	2.6	0.7	15.2
**17**	1.5	**18.1**	5.8	1.9	***16.3***	***20.2***	0.5	7.6	15.1	0.9	0.6	18.6	0.5	**17.3**	19.2	0.8	**13.8**	9.7
**18**	1.1	7.5	17.5	**14.8**	**21.5**	**27.9**	1.1	3.7	22.3	**22.8**	**25.2**	**27.9**	**26.6**	**19.1**	**33.4**	8.2	3.6	10.2
**19**	1.7	4.6	11.3	2.3	9.4	10.7	2.9	**12.0**	18.2	3.2	0.1	9.2	0.8	6.5	14.0	0.2	0.3	8.0

Figure A2S1. Standardized differences between the training sample means and species background means. The 19 BIOclim variable background means were computed for each of the six individual species. Differences were standardized for illustrative purposes (using the background standard deviation of the corresponding Bioclim variable). See Table A2S1 for variable definitions.

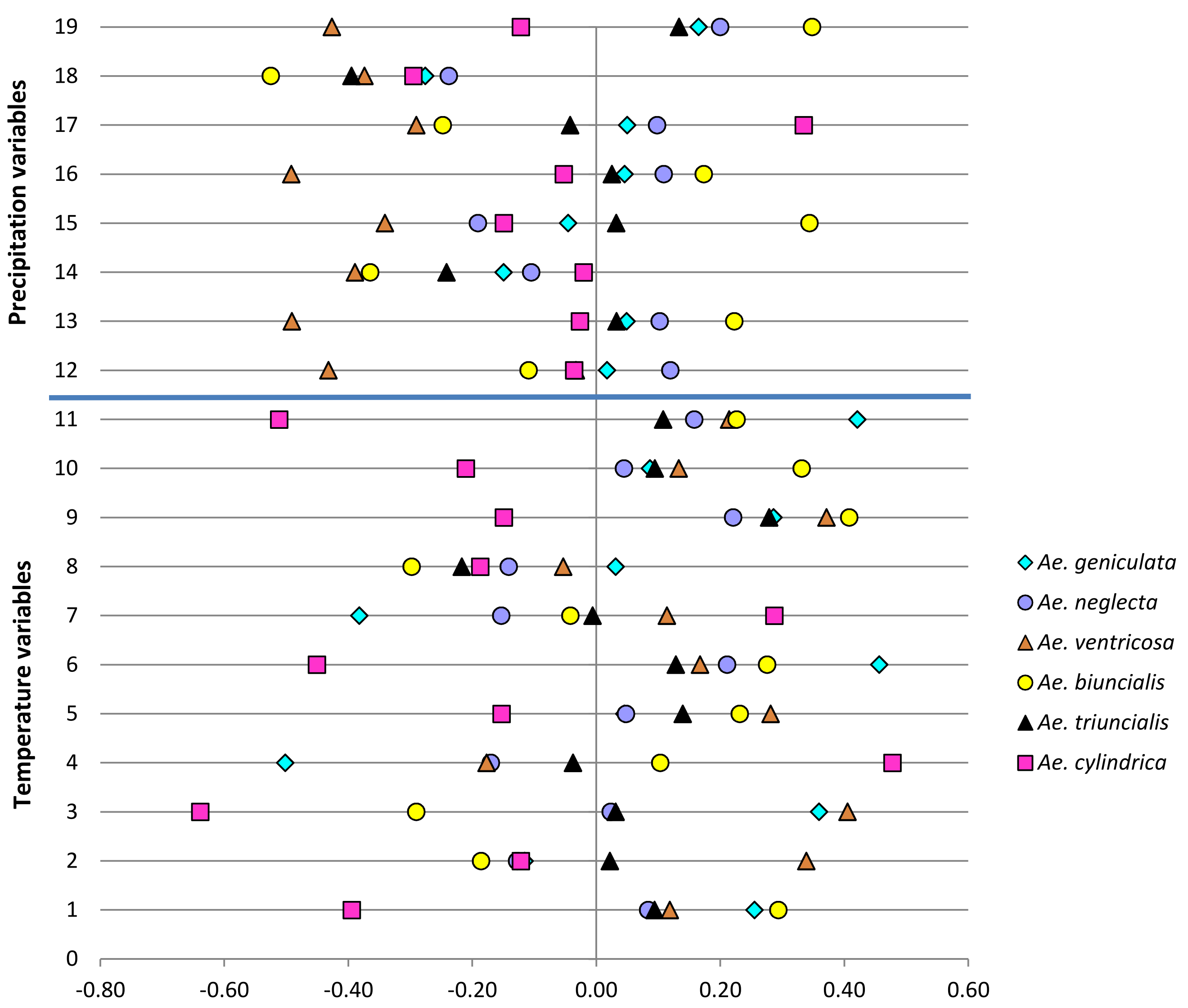

Table A2S3. Correlation matrix between BIOclim variables within the six backgrounds. As the correlation matrices were similar in all six backgrounds, we provide the average matrix. Correlation coefficients were sorted based on their absolute value. Color codes are given below. See Table A2S1 for variable definitions.

BIO	2	3	4	5	6	7	8	9	10	11	12	13	14	15	16	17	18	19
1	0.20	0.48	-0.25	0.79	0.86	-0.11	0.24	0.76	0.92	0.93	-0.47	-0.22	-0.62	0.61	-0.23	-0.61	-0.67	0.02
2		0.51	0.52	0.66	-0.23	0.81	-0.22	0.36	0.41	-0.03	-0.59	-0.49	-0.50	0.42	-0.50	-0.52	-0.49	-0.37
3			-0.44	0.36	0.43	-0.08	-0.06	0.45	0.31	0.55	-0.32	-0.22	-0.37	0.42	-0.22	-0.36	-0.41	-0.02
4				0.33	-0.66	0.91	-0.15	-0.09	0.14	-0.57	-0.30	-0.28	-0.15	0.05	-0.29	-0.20	-0.10	-0.35
5					0.40	0.51	0.05	0.74	0.94	0.55	-0.67	-0.43	-0.74	0.61	-0.45	-0.74	-0.77	-0.21
6						-0.58	0.27	0.60	0.62	0.97	-0.13	0.04	-0.35	0.37	0.03	-0.31	-0.41	0.25
7							-0.21	0.10	0.26	-0.42	-0.47	-0.42	-0.33	0.19	-0.43	-0.37	-0.29	-0.42
8								-0.23	0.17	0.25	-0.11	-0.14	0.04	-0.12	-0.15	0.04	0.20	-0.32
9									0.75	0.68	-0.47	-0.22	-0.70	0.60	-0.23	-0.66	-0.82	0.10
10										0.73	-0.60	-0.34	-0.70	0.63	-0.36	-0.70	-0.73	-0.12
11											-0.28	-0.07	-0.47	0.49	-0.08	-0.44	-0.53	0.16
12												0.85	0.75	-0.48	0.88	0.78	0.73	0.73
13													0.37	-0.04	0.99	0.39	0.40	0.88
14														-0.77	0.40	0.99	0.91	0.20
15															-0.07	-0.80	-0.71	0.08
16																0.42	0.43	0.89
17																	0.90	0.24
18																		0.11

$|r| < 0.2$	$0.2 \le |r| < 0.4$	$0.4 \le |r| < 0.6$	$0.6 \le |r| < 0.8$	$0.8 \le |r|$
