# Supplementary material for: Potential Implications of Climate Change on Aegilops Species Distribution: Sympatry of These Crop Wild Relatives with the Major European Crop Triticum aestivum and Conservation Issues
Source: PLoS One. 2016 Apr 21;11(4):e0153974. doi: 10.1371/journal.pone.0153974 (PMC4839726; doi:10.1371/journal.pone.0153974)
Supplement: S1 Fig — (PDF) [file pone.0153974.s003.pdf]

S1 Figure. Potential predicted distributions: $RCP_{4.5}$. Continuous suitability index predicted for **(A)** the current climate, **(B)** $RCP_{4.5}$ under the no migration hypothesis and **(C)** $RCP_{4.5}$ under the universal migration hypothesis. White areas correspond to cells for which the suitability index was below the species specific logistic threshold. Grey areas correspond to cells requiring extrapolation. Suitability index classes: light green [species specific threshold up to 0.55], yellow [0.55 - 0.65], and red [0.65 -1].

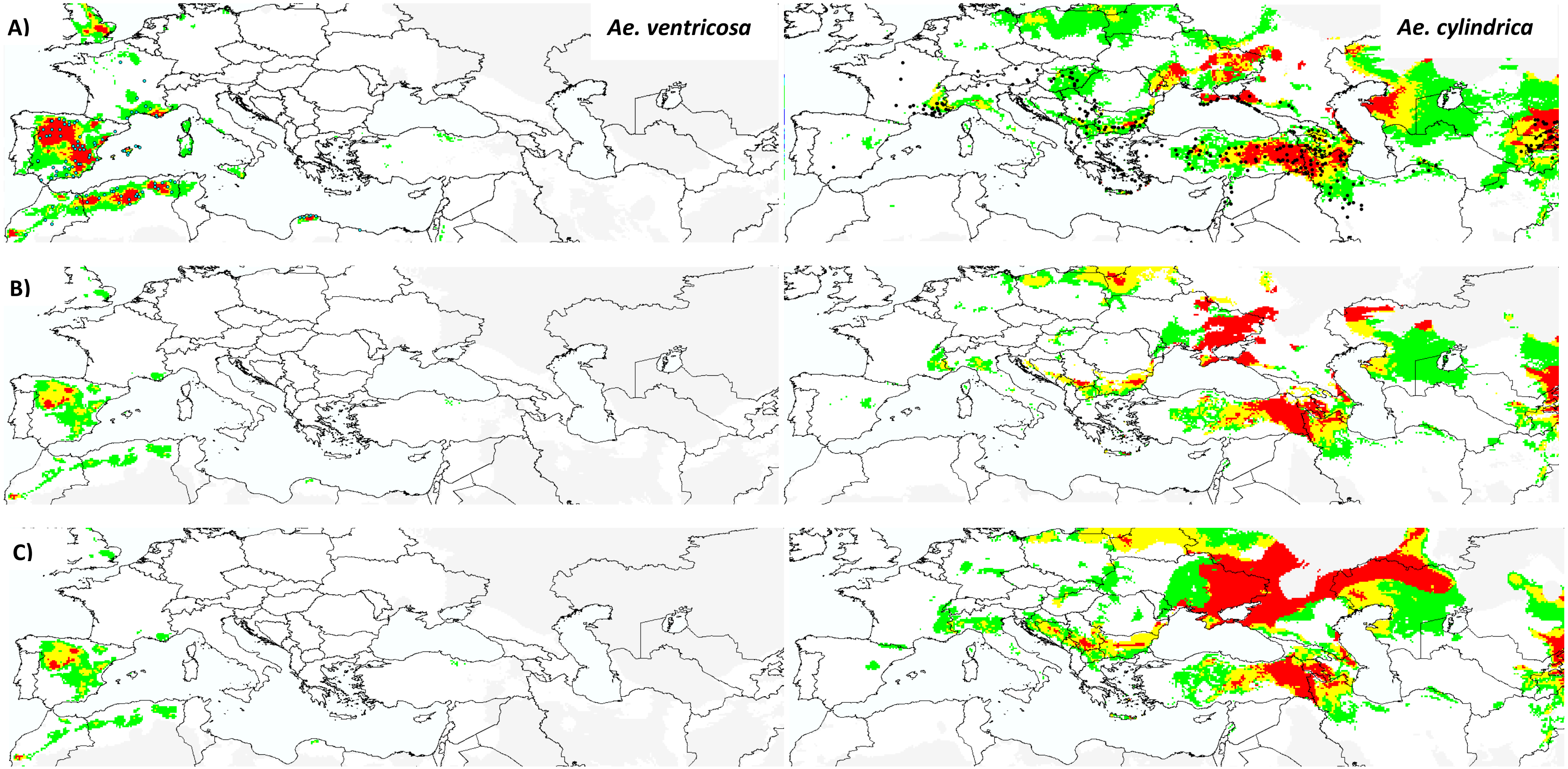

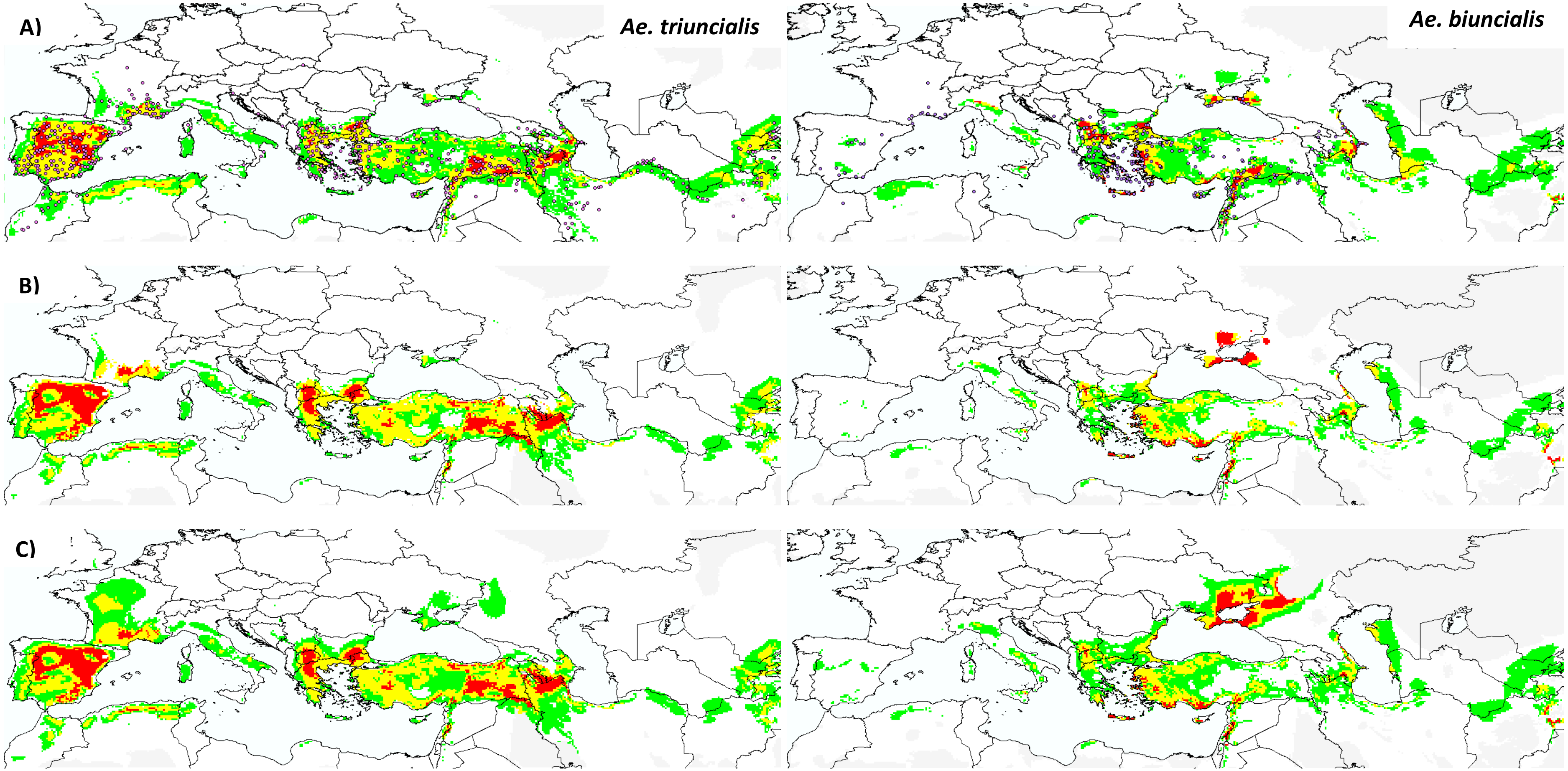
A)
Ae. triuncialis
Ae. biuncialis
B)
C)

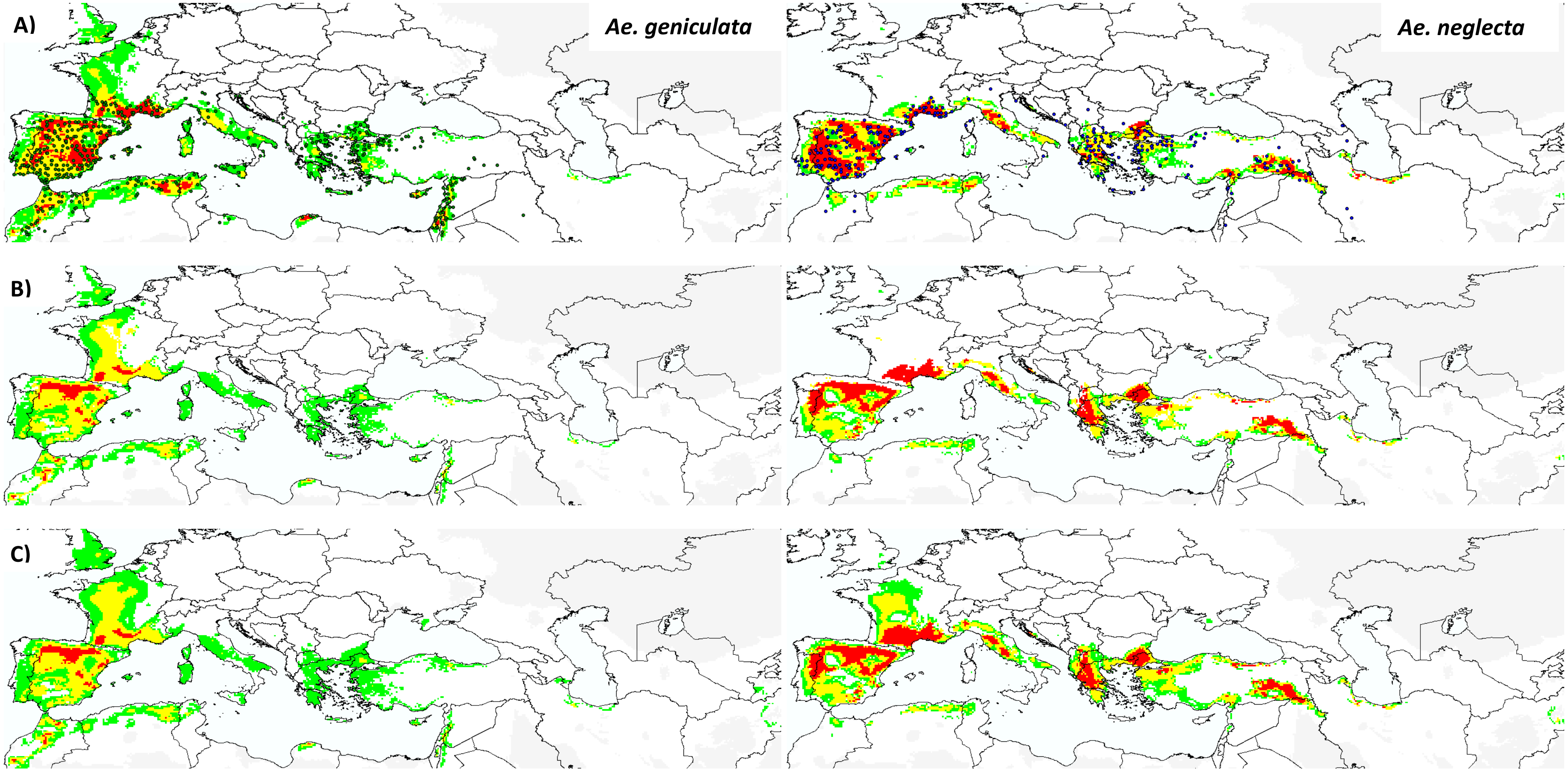
A)
Ae. geniculata
Ae. neglecta
B)
C)
