# Supplementary material for: Potential Implications of Climate Change on Aegilops Species Distribution: Sympatry of These Crop Wild Relatives with the Major European Crop Triticum aestivum and Conservation Issues
Source: PLoS One. 2016 Apr 21;11(4):e0153974. doi: 10.1371/journal.pone.0153974 (PMC4839726; doi:10.1371/journal.pone.0153974)

S3 Figure. Potential species richness: $RCP_{8.5}$. Predicted richness for **(A)** the current climate, **(B)** $RCP_{8.5}$ under the no migration hypothesis and **(C)** $RCP_{8.5}$ under the universal migration hypothesis. Light grey, dark grey, light blue, medium blue, dark blue and red correspond to cells predicted to be suitable for one to six *Aegilops* species.

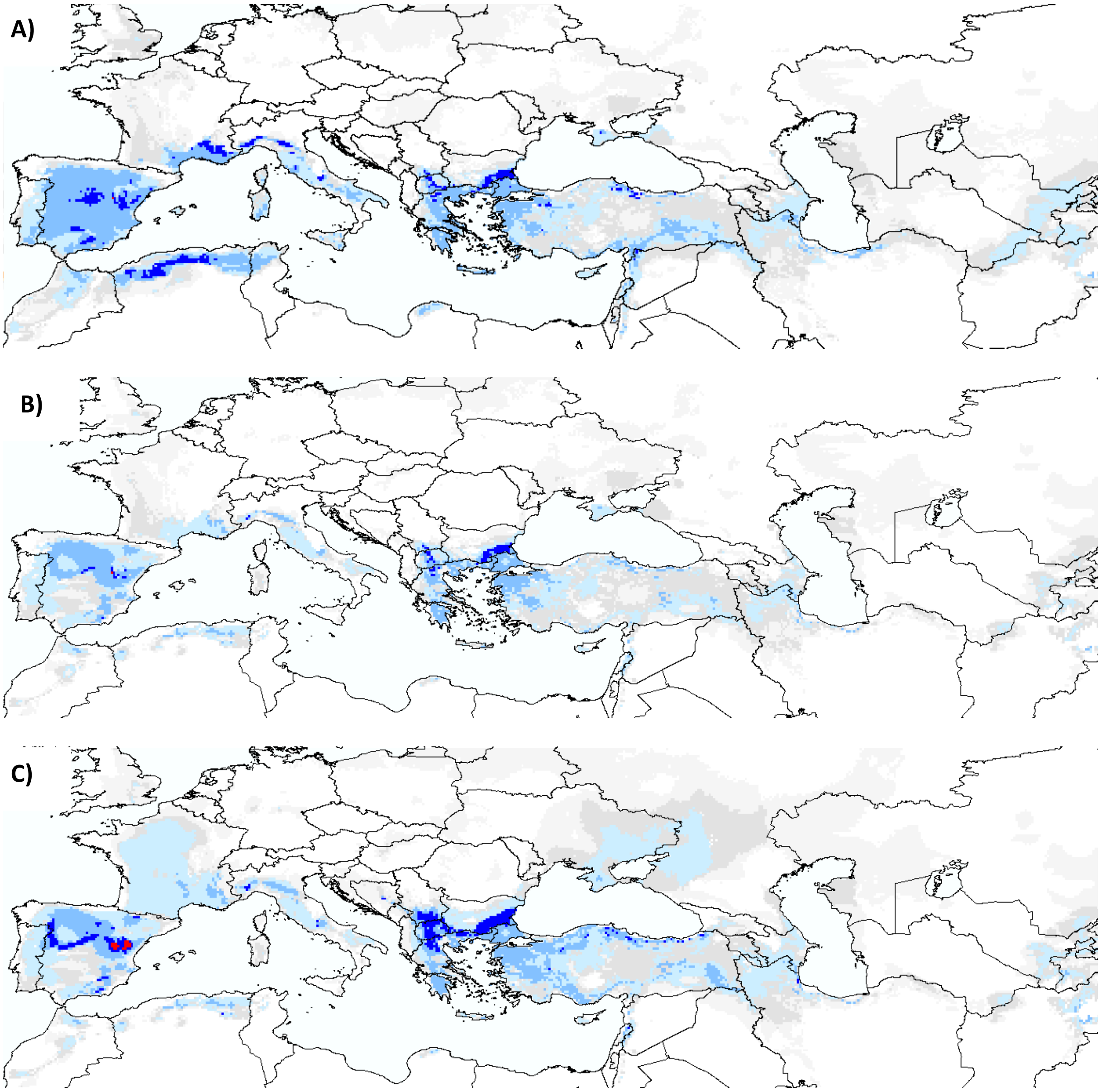

Supplement: S3 Fig — (PDF) [file pone.0153974.s005.pdf]
