# Supplementary material for: Potential Implications of Climate Change on Aegilops Species Distribution: Sympatry of These Crop Wild Relatives with the Major European Crop Triticum aestivum and Conservation Issues
Source: PLoS One. 2016 Apr 21;11(4):e0153974. doi: 10.1371/journal.pone.0153974 (PMC4839726; doi:10.1371/journal.pone.0153974)
Supplement: S4 Fig — (PDF) [file pone.0153974.s006.pdf]

S4 Figure. Most frequent potential associations of at least three species: $RCP_{8.5}$. Predicted associations for **(A)** the current climate, **(B)** $RCP_{8.5}$ under the no migration hypothesis and **(C)** $RCP_{8.5}$ under the universal migration hypothesis. All classes of potential species richness ranging from three to six are represented. The color code is given below. The frequency of each association is also reported (in % of the total number of grid cells). Boxes indicate the very most frequent associations in all RCP-by-migration hypothesis combinations.

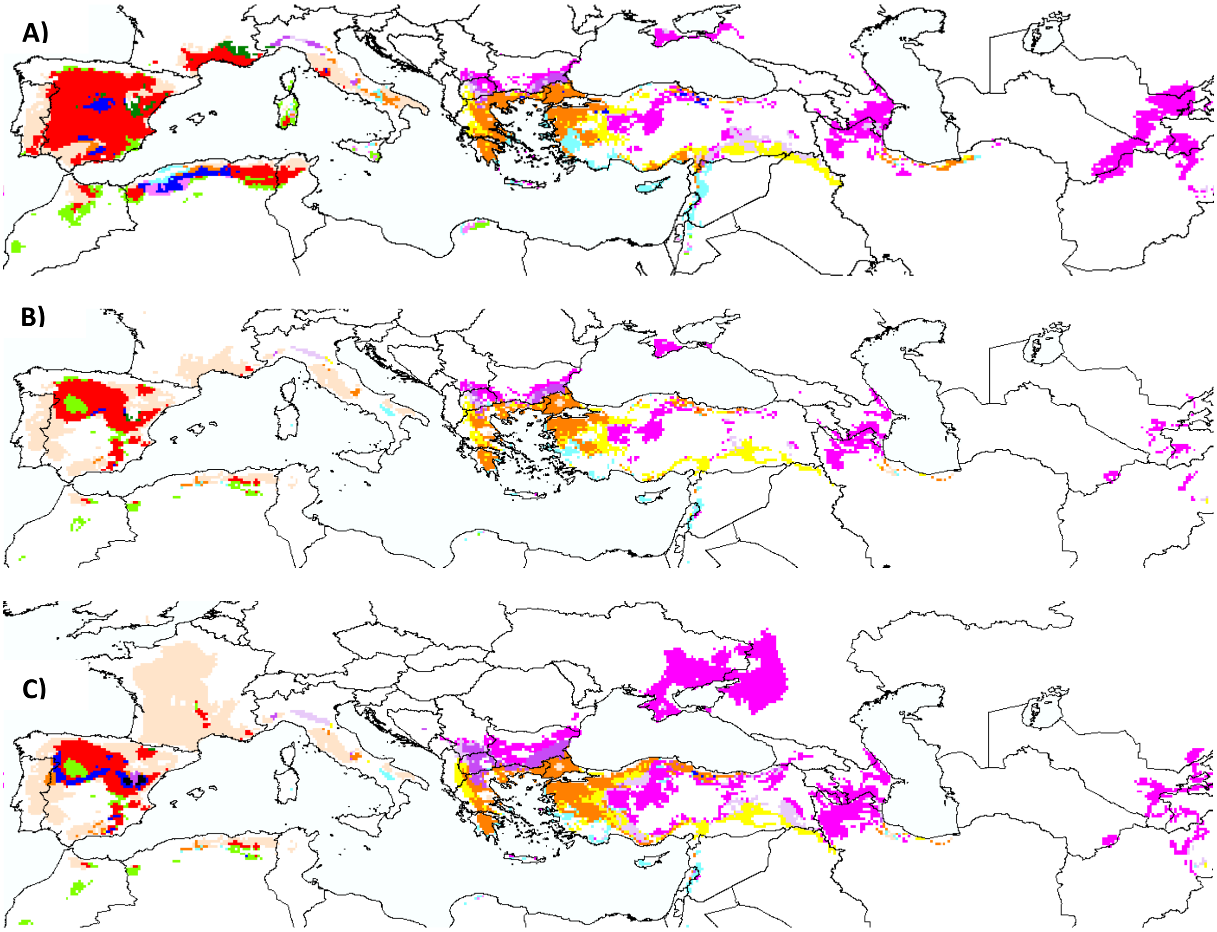

	Species						Panel		
	Ae. cyl.	*Ae. biun.*	*Ae. triun.*	*Ae. gen.*	*Ae. neg.*	*Ae. vent.*	A	B	C
	x	x	x				2.6%	1.4%	4.2%
		x	x	x			0.6%	0.3%	0.3%
		x	x		x		0.8%	0.8%	0.8%
			x	x	x		1.3%	1.9%	3.8%
			x	x		x	0.6%	0.3%	0.3%
	x	x	x		x		0.5%	0.2%	0.6%
		x	x	x	x		1.4%	0.9%	1.4%
		x	x	x		x	0.2%	0.0%	0.0%
			x	x	x	x	2.8%	1.0%	0.9%
	x	x	x	x	x		0.2%	0.1%	0.5%
	x		x	x	x	x	0.2%	0.0%	0.0%
		x	x	x	x	x	0.4%	0.0%	0.2%
	x	x	x	x	x	x	0.0%	0.0%	0.0%
