# Supplementary material for: Potential Implications of Climate Change on Aegilops Species Distribution: Sympatry of These Crop Wild Relatives with the Major European Crop Triticum aestivum and Conservation Issues
Source: PLoS One. 2016 Apr 21;11(4):e0153974. doi: 10.1371/journal.pone.0153974 (PMC4839726; doi:10.1371/journal.pone.0153974)
Supplement: S5 Fig — (PDF) [file pone.0153974.s007.pdf]

S5 Figure. Summary of changes in potential area of occupancy**.** Areas in light green remained suitable by 2050, dark green became unsuitable (losses), orange became suitable (gains). Adding light to dark green corresponds to the current binary projected predictions, light green alone to the 2050 predictions without migration and adding light green to orange to the 2050 predictions with universal migration. The grey areas correspond to cells requiring extrapolation under the current climate.

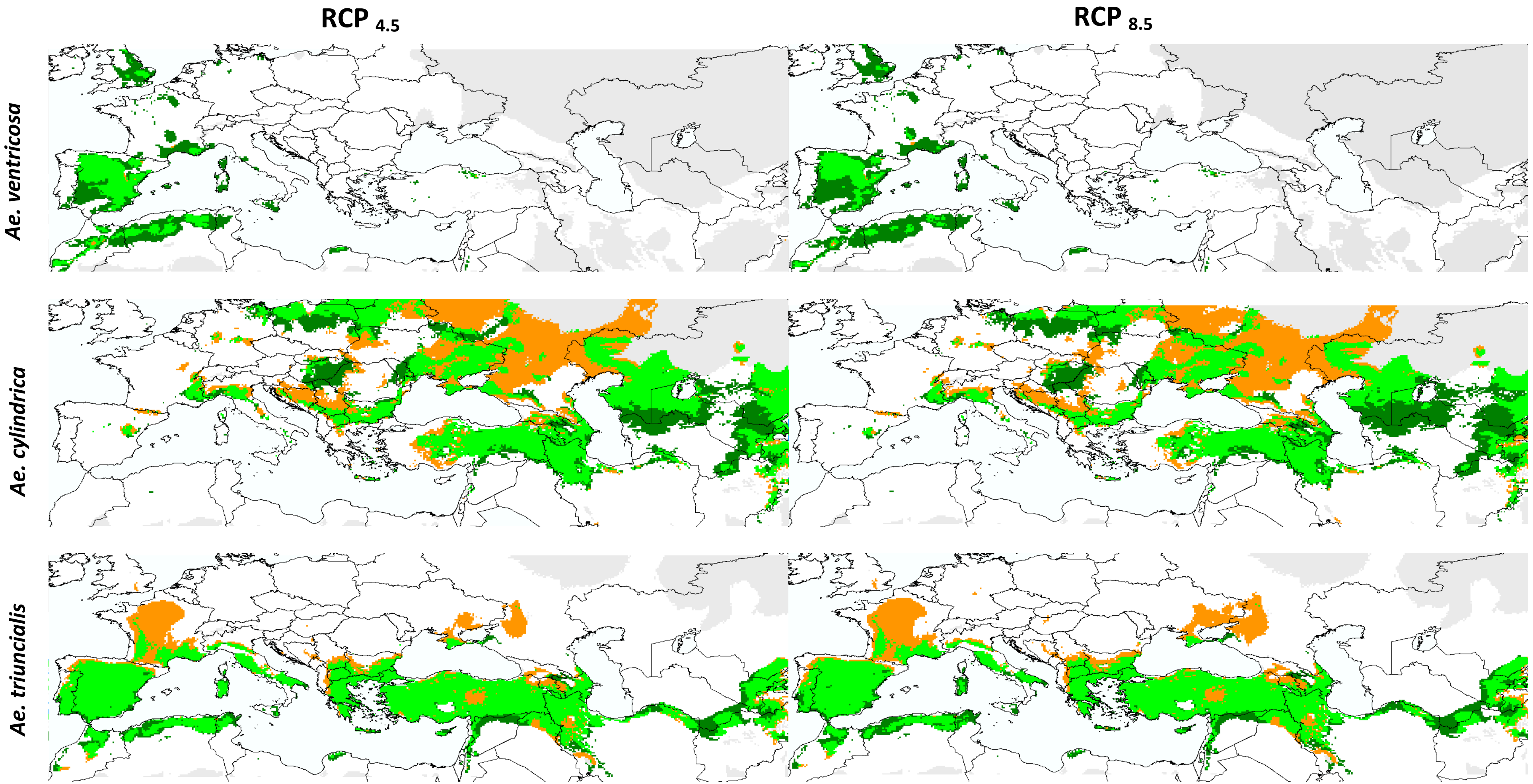

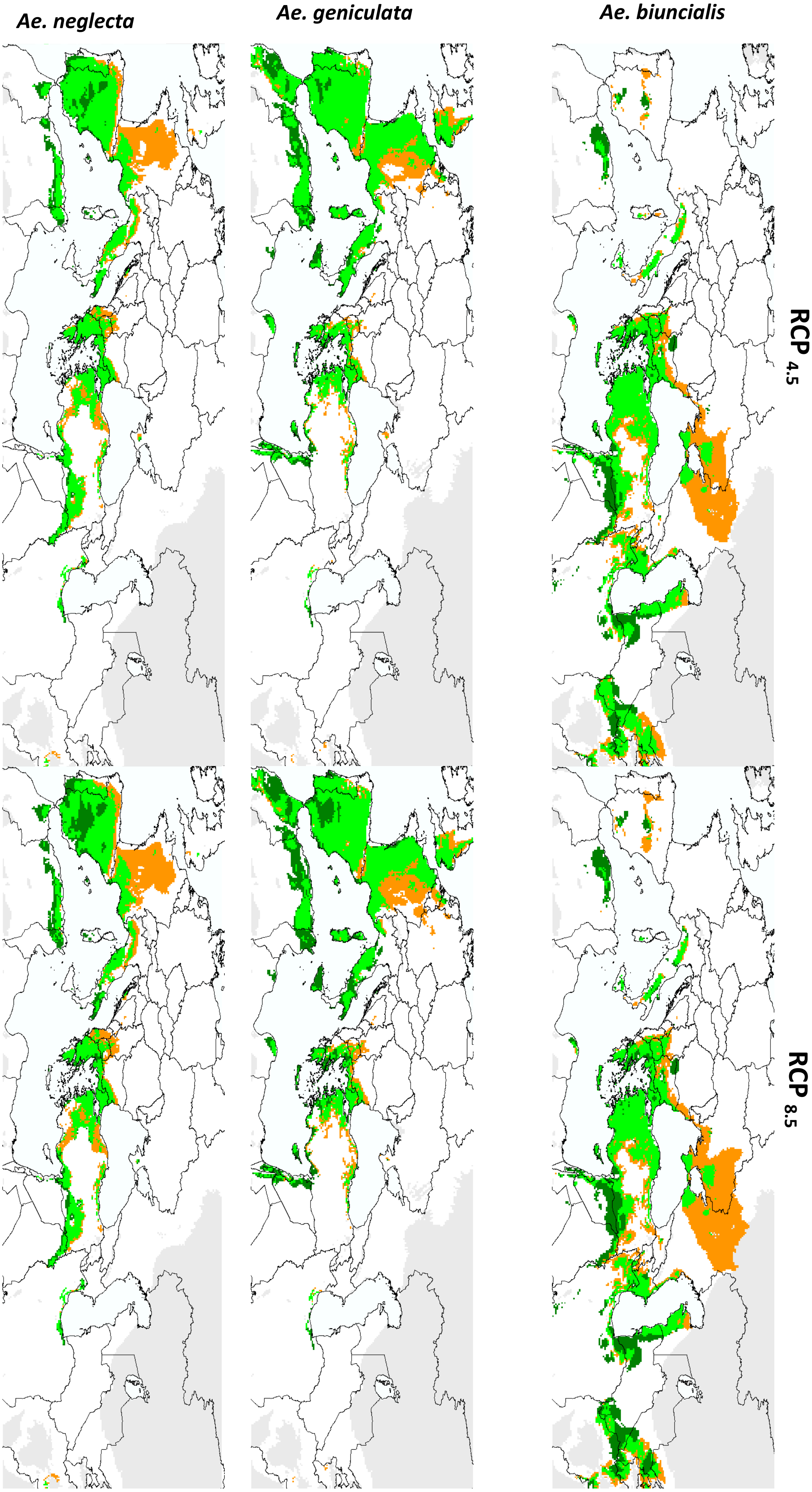
Ae. neglecta
Ae. geniculata
Ae. biuncialis
RCP 4.5
RCP 8.5
