# Supplementary material for: Potential Implications of Climate Change on Aegilops Species Distribution: Sympatry of These Crop Wild Relatives with the Major European Crop Triticum aestivum and Conservation Issues
Source: PLoS One. 2016 Apr 21;11(4):e0153974. doi: 10.1371/journal.pone.0153974 (PMC4839726; doi:10.1371/journal.pone.0153974)
Supplement: S6 Fig — (PDF) [file pone.0153974.s008.pdf]

S6 Figure. Potential sympatry index between cultivated wheat and individual *Aegilops* species in the European zone: $RCP_{4.5}$. Sympatry index for **(A)** the current climate, **(B)** $RCP_{4.5}$ under the no migration hypothesis and **(C)** $RCP_{4.5}$ under the universal migration hypothesis. PSI classes: white [0 - 0.05], grey [0.05 - 0.1], yellow [0.1 -0.15], light green [0.15 – 0.2], and dark green [0.2 - 0.27].

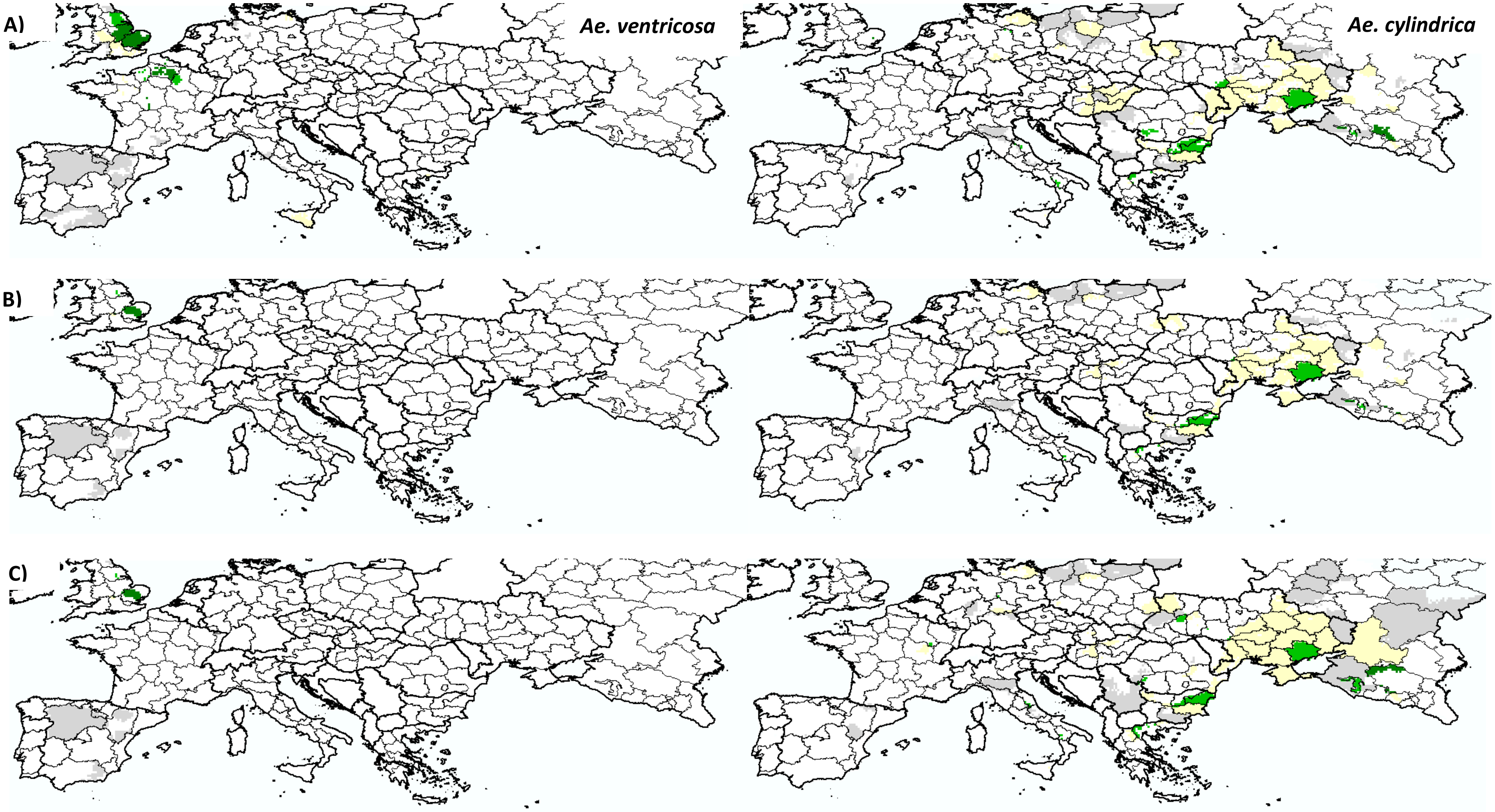

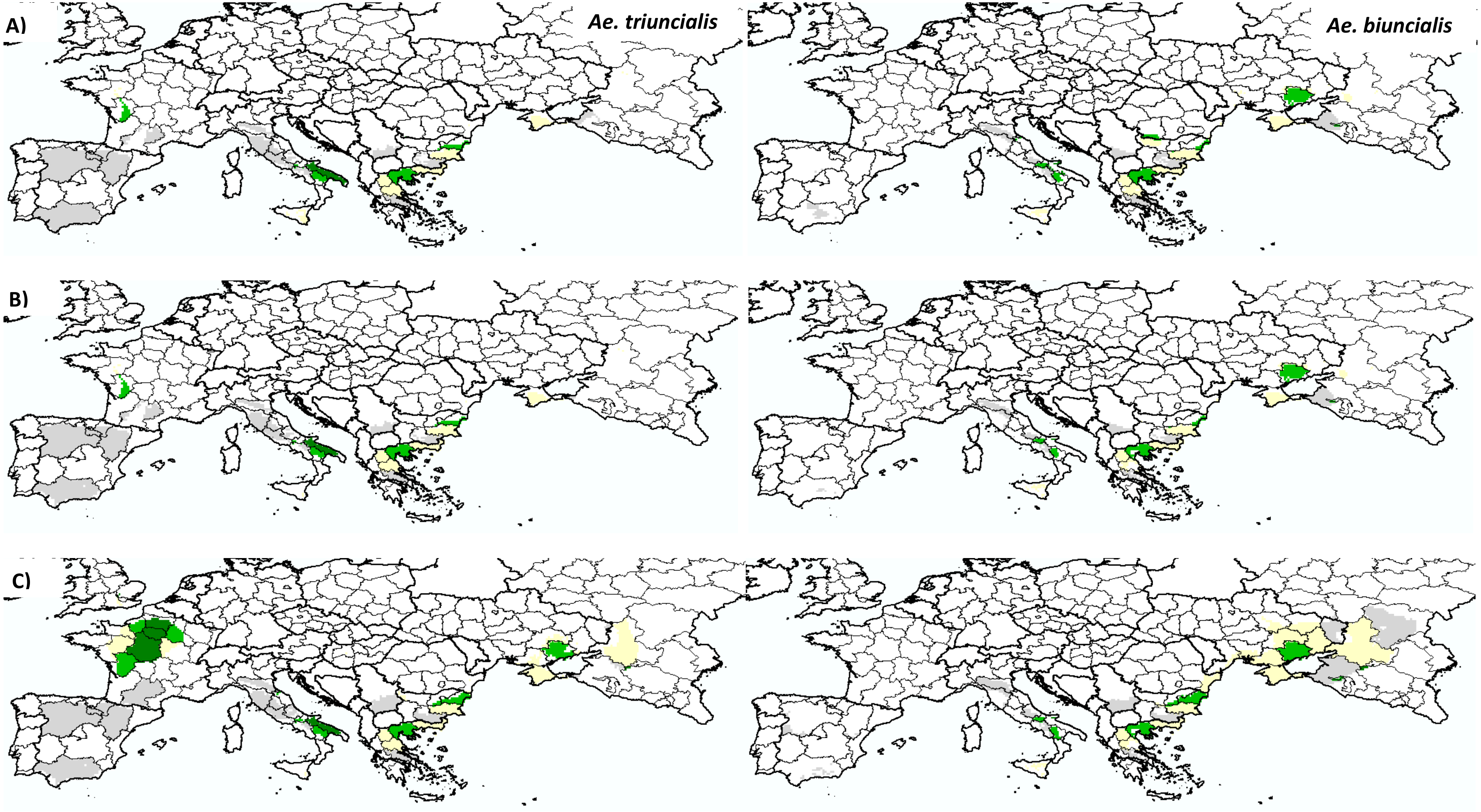
A)
Ae. triuncialis
Ae. biuncialis
B)
C)

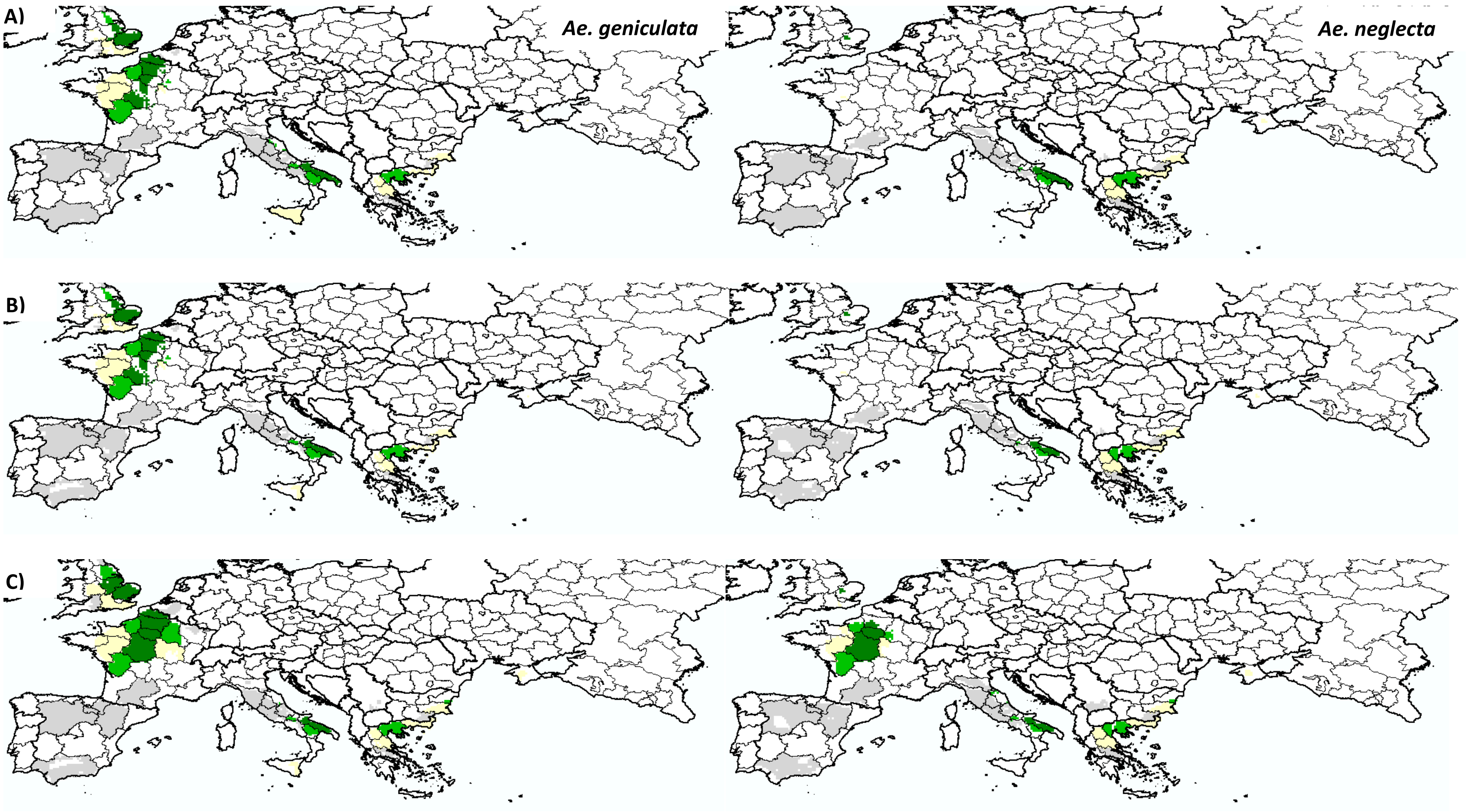

A)
Ae. geniculata
Ae. neglecta
B)
C)
