# Supplementary material for: Potential Implications of Climate Change on Aegilops Species Distribution: Sympatry of These Crop Wild Relatives with the Major European Crop Triticum aestivum and Conservation Issues
Source: PLoS One. 2016 Apr 21;11(4):e0153974. doi: 10.1371/journal.pone.0153974 (PMC4839726; doi:10.1371/journal.pone.0153974)

**S8 Figure.** Global potential sympatry index between the six *Aegilops* species and cultivated wheat in the European zone: RCP$_{8.5}$. Global sympatry index (richness x wheat proxy) for **(A)** the current climate, **(B)** RCP$_{8.5}$ under the no migration hypothesis and **(C)** RCP$_{8.5}$ under the universal migration hypothesis.

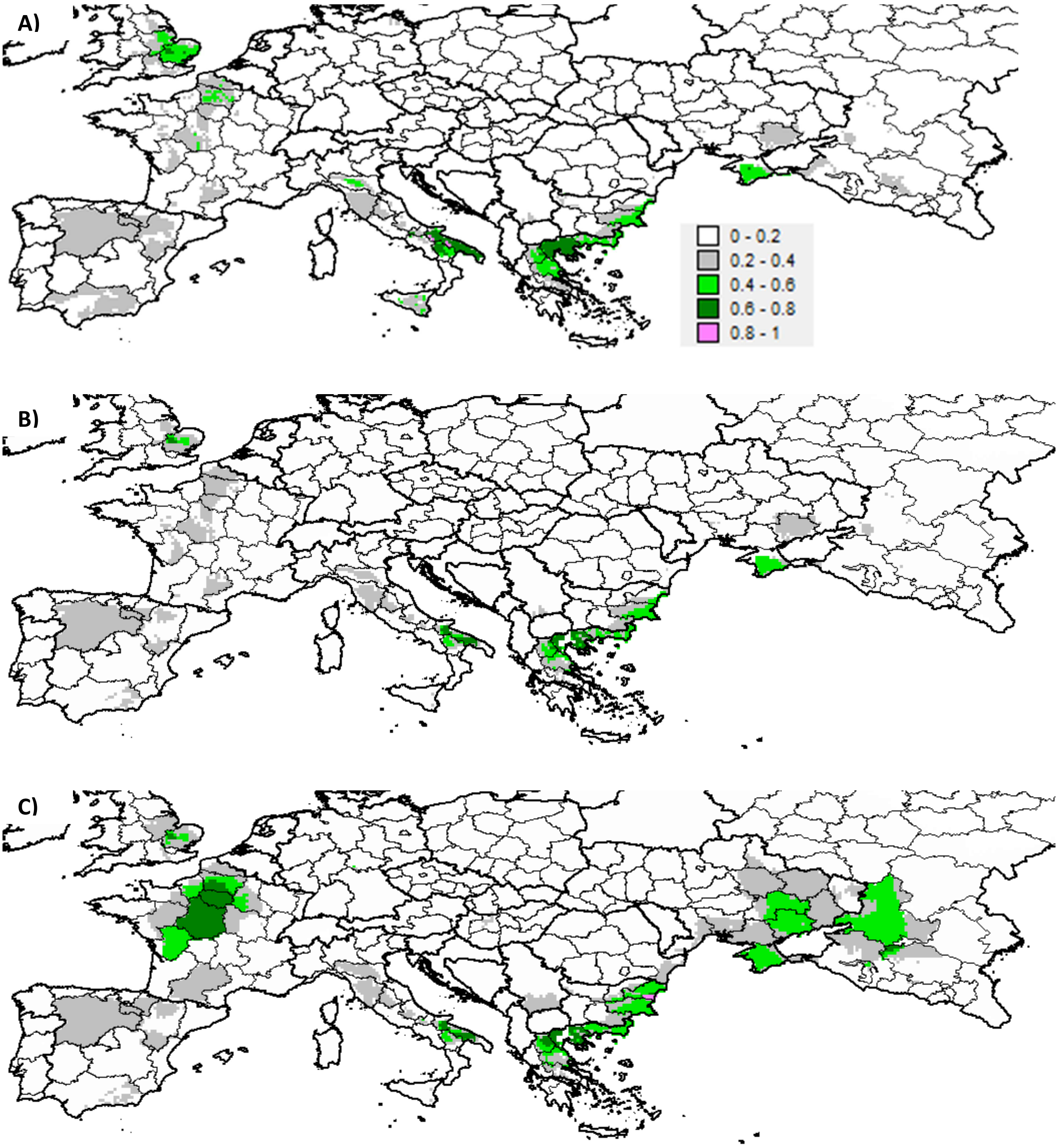

Supplement: S8 Fig — (PDF) [file pone.0153974.s010.pdf]
