# Supplementary material for: Potential Implications of Climate Change on Aegilops Species Distribution: Sympatry of These Crop Wild Relatives with the Major European Crop Triticum aestivum and Conservation Issues
Source: PLoS One. 2016 Apr 21;11(4):e0153974. doi: 10.1371/journal.pone.0153974 (PMC4839726; doi:10.1371/journal.pone.0153974)
Supplement: S1 Table — (PDF) [file pone.0153974.s012.pdf]

S1 Table. Institutions contributing to the dataset. Global Biodiversity Information Facility (GBIF) data were accessed through the data portal http://data.gbif.org, mostly in April 2013. Institutions were sorted by the resource number attributed by GBIF.

Source	*Ae. biuncialis*	*Ae. cylindrica*	*Ae.geniculata*	*Ae.neglecta*	*Ae.triuncialis*	*Ae.ventricosa*
Herbario de la Universidad de Salamanca, SALA, http://data.gbif.org/datasets/resource/239	x	x	x	x	x	x
Real Jardin Botanico ,Madrid, Vascular Plant Herbarium ,MA, http://data.gbif.org/datasets/resource/240		x	x		x	x
Herbario de la Universidad de Almeria, HUAL, http://data.gbif.org/datasets/resource/244	x		x	x	x	x
Jardín Botánico de Córdoba:, Herbarium COA, http://data.gbif.org/datasets/resource/247	x		x	x	x	x
CIBIO, Alicante, ABH-GBIF, http://data.gbif.org/datasets/resource/251			x	x	x	x
Universidad de Extremadura, UNEX, http://data.gbif.org/datasets/resource/255					x	
Herbario de la Universidad de Sevilla, SEV, http://data.gbif.org/datasets/resource/283	x					
Dirección General de Investigación, Desarrollo Tecnológico e Innovación de la Junta de Extremadura, DGIDTI, HSS, http://data.gbif.org/datasets/resource/291			x	x	x	x
Dpto de Botánica, Ecología y Fisiología Vegetal , herbario COFC, Facultad de Ciencias, Universidad de Córdoba, http://data.gbif.org/datasets/resource/292					x	
Hortus Botanicus Sollerensis Herbarium, JBS, http://data.gbif.org/datasets/resource/300				x	x	x
Herbarium Willing, BGBM, http://data.gbif.org/datasets/resource/1096	x		x	x	x	
Centre for Genetic Resources, the Netherlands, PGR passport data, NLD037, http://data.gbif.org/datasets/resource/1102			x			x
Observations du Conservatoire botanique national du Bassin parisien, CBNBP, http://data.gbif.org/datasets/resource/1103	x	x			x	x
Biologiezentrum Linz, LI, http://data.gbif.org/datasets/resource/1104			x			
United States National Plant Germplasm System Collection, USA029, http://data.gbif.org/datasets/resource/1429	x	x		x	x	x
The System-wide Information Network for Genetic Resources , SINGER, http://data.gbif.org/datasets/resource/1430	x	x	x	x	x	x
Israel Nature and Parks Authority,INPA, http://data.gbif.org/datasets/resource/1431	x		x		x	
BDBCV-General, Herbarium GJO, http://data.gbif.org/datasets/resource/1484		x	x	x	x	x
USU-UTC Specimen Database, http://data.gbif.org/datasets/resource/1508		x		x		
Institut Botanic de Barcelona, BC-Histórico, http://data.gbif.org/datasets/resource/1523	x	x	x	x	x	x
Impetus - Herbarium Hamburgense, HBG, ,http://data.gbif.org/datasets/resource/1605			x			x
IPK Genebank, http://data.gbif.org/datasets/resource/1851	x	x	x	x	x	x
SysTax - Herbaria, GI, http://data.gbif.org/datasets/resource/1875			x		x	
EURISCO, The European Genetic Resources Search Catalogue,	x	x	x	x	x	x

http://data.gbif.org/datasets/resource/1905						
Universidad de Málaga: MGC-Cormof, http://data.gbif.org/datasets/resource/8105			x	x	x	x
Herbarium Senckenbergianum , FR, http://data.gbif.org/datasets/resource/8311		x	x		x	
Polish gene bank – passport data of plants accessions which are important in human life, http://data.gbif.org/datasets/resource/8332					x	
Universidad de Oviedo. Departamento de Biología de Organismos y Sistemas, FCO-Briof, http://data.gbif.org/datasets/resource/8404					x	
Fundación Biodiversidad, Real Jardín Botánico (CSIC): Anthos. Sistema de Información de las plantas de España, http://data.gbif.org/datasets/resource/9090					x	
Cartografía de vegetación a escala de detalle 1:10.000 de la masa forestal de Andalucía, REDIAM-CMA, http://data.gbif.org/datasets/resource/10833	x	x	x	x	x	x
Nationaal Herbarium Nederland, L, http://data.gbif.org/datasets/resource/11520			x	x		x
Nationaal Herbarium Nederland, LD, http://data.gbif.org/datasets/resource/11520	x	x	x		x	
Herbarium GZU, http://data.gbif.org/datasets/resource/11968	x		x			
Tiroler Landesmuseum Ferdinandeum, TLMF, http://data.gbif.org/datasets/resource/11971		x	x	x		
Missouri Botanical Garden, MO, http://data.gbif.org/datasets/resource/12084		x				x
Catálogo Florístico Histórico de Navarra. Gobierno de Navarra, IDBD-GN, http://data.gbif.org/datasets/resource/12958			x	x	x	x
Natural History Museum, Vienna - Herbarium W, http://data.gbif.org/datasets/resource/13042		x				x
Inventario de Flora y Vegetación del Municipio de Enguídanos , Cuenca, 2010. Asociación de Desarrollo Integral de la Manchuela Conquense, ADIMAN, http://data.gbif.org/datasets/resource/13090			x	x	x	x
Universität Salzburg, SZU, http://data.gbif.org/datasets/resource/13984	x					
Sistema de Información de la vegetación Ibérica y Macaronésica, SIVIM, http://data.gbif.org/datasets/resource/14072	x		x	x	x	x
Botanical Information System of Geneva, G, http://data.gbif.org/datasets/resource/14176			x	x	x	
Inventaire National du Patrimoine Naturel, MNHN-SPN, I057 : Flore du Limousin, http://data.gbif.org/datasets/resource/14274	x		x		x	x
FlorKart - FlorenKartierung Gefaesspflanzen, BfN,http://data.gbif.org/datasets/resource/14519		x	x			
Conservatoire Botanique National Méditerranéen de Porquerolles*	x	x	x	x	x	x
Conservatoire Botanique National du Massif Central*			x		x	x
Conservatoire Botanique National Alpin*		x	x	x	x	
Conservatoire Botanique National des Pyrénées et de Midi-Pyrénées*		x	x	x	x	x
Conservatoire Botanique National Sud-Atlantique*			x		x	
M. W van Slageren (com. pers)	x	x	x	x	x	x

*Coordinates of presence points from CBNs were accessed upon special request.
