# Supplementary material for: Potential Implications of Climate Change on Aegilops Species Distribution: Sympatry of These Crop Wild Relatives with the Major European Crop Triticum aestivum and Conservation Issues
Source: PLoS One. 2016 Apr 21;11(4):e0153974. doi: 10.1371/journal.pone.0153974 (PMC4839726; doi:10.1371/journal.pone.0153974)
Supplement: S2 Table — (PDF) [file pone.0153974.s013.pdf]

S2 Table. Names of the climate models. $IPCC_5$[ǂ] 2050 data originated from 16 climate models[&] (RPC4.5 and RPC8.5 concentration pathways)[§] and were accessed through the WorldClim data portal (http://www.worldclim.org/) in April 2014.

**Model**
access1-0
bcc-csm1-1
ccsm4
cnrm-cm5
gfdl-cm3
giss-e2-r
hadgem2-ao
hadgem2-es
inmcm4
ipsl-cm5a-lr
miroc5
miroc-esm
miroc-esm-chem
mpi-esm-lr
mri-cgcm3
noresm1-m

ǂ : Intergovernmental Panel on Climate Change.

&: Data were averaged over the 16 models for each RCP as a central climate model trend was preferred over the particularities of single models (e.g. Loarie et al. 2009, doi:10.1038/nature08649; see also Knutti & Sedláček, 2012, doi:10.1038/nclimate1716).

§: Representative Concentration Pathways, Fifth Assessment Report (AR5).
