# Supplementary material for: Potential Implications of Climate Change on Aegilops Species Distribution: Sympatry of These Crop Wild Relatives with the Major European Crop Triticum aestivum and Conservation Issues
Source: PLoS One. 2016 Apr 21;11(4):e0153974. doi: 10.1371/journal.pone.0153974 (PMC4839726; doi:10.1371/journal.pone.0153974)
Supplement: S3 Table — (PDF) [file pone.0153974.s014.pdf]

S3 Table. Detailed filling estimates (%). Column headings C, 4.5 and 8.5 refer to the current, $RCP_{4.5}$ and $RCP_{8.5}$ projections; UM and NM to the universal and no migration hypothesis, respectively. Species ranking orders are given in parenthesis. The extrapolated cells columns correspond to the ratio of the remaining number of extrapolated cells predicted to be suitable over the total number of cells predicted to be suitable. The countries where extrapolated cells were located were named after their ISO3 code: IRN, IRQ, KAZ, RUS and UKR stands for Iran, Iraq, Kazakhstan, Russia and Ukraine, respectively. For instance, once extrapolated cells were removed from the current projection, *Ae. cylindrica* had 6.8% and 6.9% of its remaining suitable cells resulting from extrapolation in $RCP_{4.5}$ and $RCP_{8.5}$ projection, respectively.

**Full grid**						**Extrapolated cells**							
		**UM**		**NM**		**UM 4.5**				**UM 8.5**			
**Species**	**C**	**4.5**	**8.5**	**4.5**	**8.5**	**RUS**	**KAZ**	**UKR+IRN**	**IRN+IRQ**	**RUS**	**KAZ**	**UKR+IRN**	**IRN+IRQ**
Ae. biuncialis	$11.0_{(4)}$	$13.7_{(3)}$	$13.8_{(3)}$	$8.1_{(4)}$	$7.0_{(4)}$	4.9%		0.2%		5.1%		0.2%	
Ae. cylindrica	$23.9_{(1)}$	$27.8_{(1)}$	$26.5_{(1)}$	$16.5_{(1)}$	$14.9_{(1)}$	6.3%	0.4%	0.1%		6.4%	0.3%	0.1%	
Ae. geniculata	$12.3_{(3)}$	$12.3_{(4)}$	$11.4_{(4)}$	$10.0_{(3)}$	$8.6_{(3)}$								
Ae. neglecta	$8.4_{(5)}$	$10.2_{(5)}$	$9.7_{(5)}$	$7.0_{(5)}$	$6.2_{(5)}$								
Ae. triuncialis	$18.7_{(2)}$	$20.4_{(2)}$	$20.6_{(2)}$	$15.6_{(2)}$	$14.8_{(2)}$				0.8%				1.3%
Ae. ventricosa	$6.3_{(6)}$	$2.5_{(6)}$	$1.6_{(6)}$	$2.5_{(6)}$	$1.6_{(6)}$								

**European zone**	**Within full grid**					**Alone**				
		**UM**		**NM**			**UM**		**NM**	
**Species**	**C**	**4.5**	**8.5**	**4.5**	**8.5**	**C**	**4.5**	**8.5**	**4.5**	**8.5**
Ae. biuncialis	$2.6_{(6)}$	$5.9_{(5)}$	$7.0_{(4)}$	$2.1_{(5)}$	$2.0_{(5)}$	$5.6_{(6)}$	$12.7_{(5)}$	$15.0_{(4)}$	$4.6_{(5)}$	$4.3_{(5)}$
Ae. cylindrica	$9.4_{(1)}$	$15.7_{(1)}$	$16.0_{(1)}$	$6.5_{(2)}$	$6.4_{(2)}$	$20.1_{(1)}$	$33.8_{(1)}$	$34.4_{(1)}$	$13.9_{(2)}$	$13.7_{(2)}$
Ae. geniculata	$8.2_{(2)}$	$9.3_{(2)}$	$8.8_{(3)}$	$7.4_{(1)}$	$6.7_{(1)}$	$17.6_{(2)}$	$20.0_{(2)}$	$19.0_{(3)}$	$16.0_{(1)}$	$14.4_{(1)}$
Ae. neglecta	$5.4_{(4)}$	$7.2_{(4)}$	$6.8_{(5)}$	$4.7_{(4)}$	$4.1_{(4)}$	$11.5_{(4)}$	$15.4_{(4)}$	$14.7_{(5)}$	$10.0_{(4)}$	$8.9_{(4)}$
Ae. triuncialis	$6.4_{(3)}$	$9.3_{(3)}$	$10.1_{(2)}$	$5.9_{(3)}$	$5.6_{(3)}$	$13.8_{(3)}$	$20.0_{(3)}$	$21.6_{(2)}$	$12.7_{(3)}$	$12.1_{(3)}$
Ae. ventricosa	$4.1_{(5)}$	$1.8_{(6)}$	$1.3_{(6)}$	$1.8_{(6)}$	$1.2_{(6)}$	$8.8_{(5)}$	$3.8_{(6)}$	$2.7_{(6)}$	$3.8_{(6)}$	$2.6_{(6)}$

**Non-European zone**	**Within full grid**					**Alone**				
		**UM**		**NM**			**UM**		**NM**	
**Species**	**C**	**4.5**	**8.5**	**4.5**	**8.5**	**C**	**4.5**	**8.5**	**4.5**	**8.5**
Ae. biuncialis	$8.4_{(3)}$	$7.8_{(3)}$	$6.9_{(3)}$	$6.0_{(3)}$	$5.0_{(3)}$	$15.8_{(3)}$	$14.5_{(3)}$	$12.8_{(3)}$	$11.1_{(3)}$	$9.4_{(3)}$
Ae. cylindrica	$14.5_{(1)}$	$12.1_{(1)}$	$10.4_{(2)}$	$10.1_{(1)}$	$8.5_{(2)}$	$27.1_{(1)}$	$22.6_{(1)}$	$19.5_{(2)}$	$18.8_{(1)}$	$15.9_{(2)}$
Ae. geniculata	$4.2_{(4)}$	$3.0_{(5)}$	$2.5_{(5)}$	$2.5_{(4)}$	$2.0_{(4)}$	$7.8_{(4)}$	$5.5_{(5)}$	$4.7_{(5)}$	$4.7_{(4)}$	$3.7_{(5)}$
Ae. neglecta	$3.0_{(5)}$	$3.0_{(4)}$	$2.8_{(4)}$	$2.3_{(5)}$	$2.1_{(5)}$	$5.6_{(5)}$	$5.7_{(4)}$	$5.3_{(4)}$	$4.4_{(5)}$	$3.9_{(4)}$
Ae. triuncialis	$12.3_{(2)}$	$11.1_{(2)}$	$10.6_{(1)}$	$9.7_{(2)}$	$9.2_{(1)}$	$23.0_{(2)}$	$20.7_{(2)}$	$19.8_{(1)}$	$18.2_{(2)}$	$17.1_{(1)}$
Ae. ventricosa	$2.2_{(6)}$	$0.8_{(6)}$	$0.4_{(6)}$	$0.7_{(6)}$	$0.4_{(6)}$	$4.1_{(6)}$	$1.4_{(6)}$	$0.7_{(6)}$	$1.4_{(6)}$	$0.7_{(6)}$
